# Preadaptation and post-introduction evolution facilitate the invasion of *Phragmites australis* in North America

**DOI:** 10.1002/ece3.1286

**Published:** 2014-11-25

**Authors:** Wen-Yong Guo, Carla Lambertini, Loc Xuan Nguyen, Xiu-Zhen Li, Hans Brix

**Affiliations:** 1 Department of Bioscience, Aarhus UniversityOle Worms Allé 1, 8000, Aarhus C, Denmark; 2 State Key Laboratory of Estuarine and Coastal Research, East China Normal UniversityShanghai, 200062, China; 3 College of Environment and Natural Resources, Campus II, Can Tho University3/2 Street, Ninh Kieu District, Can Tho City, Vietnam

**Keywords:** Biomass allocation, common reed, common-environment experiment, discriminant analysis, ecophysiological trade-off, functional traits, invasion ecology, leaf construction cost, photosynthesis, standardized major axis (SMA)

## Abstract

Compared with non-invasive species, invasive plant species may benefit from certain advantageous traits, for example, higher photosynthesis capacity and resource/energy-use efficiency. These traits can be preadapted prior to introduction, but can also be acquired through evolution following introduction to the new range. Disentangling the origins of these advantageous traits is a fundamental and emerging question in invasion ecology. We conducted a multiple comparative experiment under identical environmental condition with the invasive haplotype M lineage of the wetland grass *Phragmites australis* and compared the ecophysiological traits of this invasive haplotype M in North America with those of the European ancestor and the conspecific North American native haplotype E lineage, *P. australis* ssp. *americanus*. The invasive haplotype M differed significantly from the native North American conspecific haplotype E in several ecophysiological and morphological traits, and the European haplotype M had a more efficient photosynthetic apparatus than the native North American *P. australis* ssp. *americanus*. Within the haplotype M lineage, the introduced North American *P. australis* exhibited different biomass allocation patterns and resource/energy-use strategies compared to its European ancestor group. A discriminant analysis of principal components separated the haplotype M and the haplotype E lineages completely along the first canonical axis, highly related to photosynthetic gas-exchange parameters, photosynthetic energy-use efficiency and payback time. The second canonical axis, highly related to photosynthetic nitrogen use efficiency and construction costs, significantly separated the introduced *P. australis* in North America from its European ancestor. *Synthesis*. We conclude that the European *P. australis* lineage was preadapted to be invasive prior to its introduction, and that the invasion in North America is further stimulated by rapid post-introduction evolution in several advantageous traits. The multicomparison approach used in this study could be an effective approach for distinguishing preadaptation and post-introduction evolution of invasive species. Further research is needed to link the observed changes in invasive traits to the genetic variation and the interaction with the environment.

## Introduction

The mechanisms that underlie plant invasions are complex, and no individual mechanism can sufficiently explain biological invasions (Nentwig 2007; Bennett et al. 2011; Larkin et al. 2011; Qin et al. 2013). Invasive mechanisms can be invasion stage and/or species dependent (Zedler and Kercher 2004; Pyšek et al. 2009; Gurevitch et al. 2011). For example, native-range distributional characteristics can be dominant in the early stage of the invasion, while biological traits^1^ are more important in later stages of invasions (Nentwig 2007; Pyšek et al. 2009). Species with a large native geographical range generally have the ability to survive under a wide range of environmental conditions, and as a consequence also have a suit of preadapted traits allowing them to establish in new ranges (Pyšek and Richardson 2007; Bucharova and van Kleunen 2009; Pyšek et al. 2009; van Kleunen et al. 2010b, 2011). In addition, after the introduction to new areas, the invasion can be stimulated by rapid adaptive changes in advantageous traits (Pyšek et al. 2009; Henery et al. 2010). In a recent review of 53 invasive species, Parker et al. (2013) found that nearly half of the studied species performed similarly across the introduced and native ranges, while the others exhibited traits that performed better in the introduced range, suggesting that evolutionary adaptive changes did occur after introduction to a new range.

Compared to non-invasive species, plant invaders tend to have a higher photosynthetic capacity, a higher specific leaf area, higher nitrogen contents and lower leaf construction costs (Griffin 1994; Feng et al. 2007b, 2011; Leishman et al. 2007; Zou et al. 2007; Mozdzer and Zieman 2010; Osunkoya et al. 2010; Li et al. 2011; Heberling and Fridley 2013), even though contradictory results have been reported (McDowell 2002; Feng et al. 2007a,b; Heberling and Fridley 2013). As discussed by Hierro et al. (2005), Schlaepfer et al. (2010) and Parker et al. (2013), the majority of these trait-comparison studies are, however, based on common-environment experiments with invasive species and co-occurring native congeners (e.g., Feng et al. 2007a,b; van Kleunen et al. 2010b; Mozdzer and Zieman 2010; Kumschick et al. 2013). While these studies are fundamental to identify the advantageous traits of the invader, they are inadequate to pinpoint the source of such advantages, that is, whether the invasive traits are preadapted, or evolved post-introduction (Richardson and Pyšek 2006; Keller and Taylor 2008; van Kleunen et al. 2010a,b; Riis et al. 2010; Schlaepfer et al. 2010; Atwood and Meyerson 2011; Bennett et al. 2011; Gurevitch et al. 2011). Meanwhile, an increasing number of studies has focused on trait differences between invasive populations and their source populations in the native range (Bossdorf et al. 2005; Barrett et al. 2008; Keller and Taylor 2008; Whitney and Gabler 2008; Schlaepfer et al. 2010; Alexander et al. 2012). This ancestor-descendent comparison (Keller and Taylor 2008) is suitable for detecting post-introduction evolution, but does not address preadapted traits. Only multiple comparisons consisting of the aforementioned two types of comparisons simultaneously, that is, comparison of the invader with its congeneric populations in the introduced range, and the ancestor-descendent comparison of the invader, can disentangle the contributions of prior evolutionary history and post-introduction evolution to invasion. In addition, comparisons of the respective relationships of ancestor and descendent of the invader with the native congener of the invader can give additional information about the post-introduction evolution and the trade-offs during the invasion process.

*Phragmites australis* (Cav.) Trin. ex Steud. is one of the most widespread perennial grasses in the world (Clevering and Lissner 1999). Although *P. australis* is native to North America, its recent rapid spread has been attributed to a nonnative haplotype (Haplotype M) of *P. australis* that is reported to be introduced from Eurasia in the late 1700s or early 1800s (Saltonstall 2002). This nonnative *P. australis* lineage has now dramatically altered the composition and functionality of many estuarine and freshwater wetland communities throughout North America, particularly along the Atlantic coast and in the Chesapeake Bay region (Saltonstall 2002; Meyerson et al. 2009, 2012; Mozdzer and Zieman 2010; Guo et al. 2013). The introduced *P. australis* exhibits many characteristics of a successful invasive species, such as rapid growth, high biomass, rhizome fragmentation, and tolerance to high salinities (Meyerson et al. 2009). Furthermore, Mozdzer and Zieman (2010) demonstrated that the invasive lineage displays a higher rate of photosynthesis, a higher stomatal conductance and a higher specific leaf area than the native North American lineage. The cited studies successfully explain the invasiveness of this Eurasian *P. australis* in North America but not the origin of the invasive characteristics.

A global collection of *Phragmites* genotypes from all continents is cultured in a common-environment setting at Aarhus University, Denmark (Lambertini et al. 2006). We used a subset of *Phragmites* genotypes from this collection to investigate general questions about preadaptation and post-introduction evolution within this species. We chose genotypes on a biogeographic scale to compare the ecophysiological traits of the invasive *P. australis* in North America with those of the European ancestor and the conspecific North American native *P. australis* ssp. *americanus*. The hypotheses tested were: (1) the native-range haplotype M has some advantageous traits compared to the native North American congener, which make it preadapted to be invasive; and (2) the introduced haplotype M exhibits divergent traits compared with its ancestor because of post-introduction evolution during the process of invasion in North America.

## Materials and Methods

### Plant material

The study was conducted outdoors in the experimental *Phragmites* garden at Aarhus University (56°13′N, 10°07′E, approximately 64 m above sea level), located north of Aarhus city, on the east side of the peninsula of Jutland, Denmark. The location has a temperate oceanic climate with an average temperature of 0°C in the coldest month (February) and 17°C in the warmest month (July). The mean annual precipitation is approximately 700 mm, without a dry season. During summer, there are up to 18 h of daylight per day.

The plants in the *Phragmites* garden were obtained from rhizomes collected in the field from geographically distant stands of *P. australis*. Each genotype was kept in its own 0.65-m diameter pot, containing a mixture of a commercial soil and quartz sand (approximately 50% of each). The pots were half-buried into the ground with the soil surface at level with the surrounding ground and placed at a distance of about 2 m between pots in an open area without shade from trees or other structures. The plants were watered with phreatic water every second day and fertilized with a commercial NPK fertilizer with micronutrients once a week (Lambertini et al. 2012b). To minimize the potential stress from a pot bound root mass, the plants were replanted every third year using a fraction of the root system. The *Phragmites* genotypes used in this study had been grown in the garden since 2001 under these conditions and displayed a comparable vegetative development throughout the years.

We chose genotypes from temperate Europe as the native source population of the invasive *P. australis*, as the most likely origin of this invasive lineage is temperate Europe (Plut et al. 2011; Lambertini et al. 2012b). In the introduced range, we chose genotypes of the invasive lineage collected along the East Coast of North America, as the New York City area has been shown to be the landing site of this lineage (Saltonstall 2002). The native North American conspecific *P. australis* ssp. *americanus* selected for the study also came from this region. The genotypes were sequenced (Lambertini et al. 2012a,b) following Saltonstall (2002). The invasive *P. australis* lineage belongs to haplotype M (defined by NCBI accession numbers AY016335 for the *trn*T-*trn*L region and AY016327 for the *rbc*L-*psa*I region), and the native North American conspecific *P. australis ssp. americanus* belongs to haplotype E (defined by NCBI accession numbers AY016325 for the *trn*T-*trn*L region and AY016333 for the *rbc*L-*psa*I region). In total, we included six genotypes of the native European population of the invasive haplotype M (hereafter denoted the EU group), nine genotypes of the introduced haplotype M population in North America (hereafter denoted the AM group), and five genotypes of the native North American conspecific *P. australis* ssp. *americanus* lineage (hereafter denoted the AMn group) in our analyses (Table 1).

**Table 1:** Origin and haplotype ID of the 20 genotypes used in this study.

Genotypes	Country, state	Coordinates	Haplotype
AM 115	US, Maryland	38°46′18″N, 76°04′58″W	M
AM 152	Canada, Quebec	45°34′00″N, 73°50′60″W	M
AM 114	US, Ohio	41°33′46″N, 83°39′14″W	M
AM 206	US, Connecticut	41°13′20″N, 73°03′25″W	M
AM 199	US, Massachusetts	42°29′26″N, 71°16′36″W	M
AM 99	US, North Carolina	36°16′12″N, 77°35′25″W	M
AM 180	US, Delaware	39°34′30″N, 75°42′25″W	M
AM 191	US, New York	43°16′35″N, 77°16′40″W	M
AM 186	US, Virginia	37°17′11″N, 75°55′22″W	M
EU 67	Belgium	51°13′00″N, 04°25′00″E	M
EU 639	Germany	51°49′00″N, 13°49′00″E	M
EU 85	Lithuania	55°20′56″N, 21°28′59″E	M
EU 801	Switzerland	47°13′08″N, 08°41′37″E	M
EU 163	Holland	51°19′44″N, 04°08′57″E	M
EU 172	Slovenia	46°03′19″N, 14°30′52″E	M
AMn 204	Canada, Manitoba	49°58′00″N, 98°17′60″W	E
AMn 55	US, Minnesota	46°52′26″N, 96°46′02″W	E
AMn 130	Canada, Manitoba	49°58′00″N, 98°17′60″W	E
AMn 65	US, Michigan	41°47′07″N, 83°22′25″W	E
AMn 211	US, Minnesota	44°00′02″N, 96°19′02″W	E

### Morphological traits

The five tallest shoots of each genotype were measured on May 30, 2011. We measured the height from the tip of the shoot to the ground and the basal diameter of each shoot in the field. Subsequently, the five shoots were cut and weighed to obtain the fresh shoot mass. The youngest fully expanded leaf per shoot was cut off, weighed to obtain the fresh leaf mass, and the length and width at the widest point of the leaf were measured. The leaf area was determined using a Li-3000C leaf area meter (LI-COR Inc., Lincoln, NE). The leaf and the entire shoot were then oven-dried at 105°C for 2 h, followed by drying at 60°C for at least 48 h for dry mass determination. The dried shoots were separated into leaf blades, leaf sheaths and stems, and weighed for biomass allocation determination.

The specific leaf area (SLA) was calculated as the ratio of the leaf area to the leaf dry mass. The leaf thickness was calculated according to Vile et al. (2005):


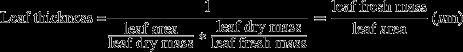
(1)

### Leaf gas exchange

The leaf gas-exchange rates were measured in situ on the youngest healthy, fully developed leaves of five shoots per genotype using a portable photosynthesis system (Li-6400XT; LI-COR Inc., Lincoln, NE) equipped with CO_2_- and temperature-control modules on clear days between 10:00 and 16:00 during the period June 27–July 11, 2011. The airflow through the leaf chamber was set to 400 *μ*mol·s^−1^, the chamber temperature to 28°C and the CO_2_ concentration to 400 *μ*mol·mol^−1^. The light-saturated photosynthetic capacity (*A*) was measured at a photosynthetic photon flux density (PPFD) of 2000 *μ*mol·photons·m^−2^·s^−1^ provided by a blue-red LED light source mounted above the leaf cuvette. The lamp was switched off and the chamber darkened (0 *μ*mol·photon·m^−2^·s^−1^) to measure dark respiration (*R*). Each reading was logged after a 3–5 min period of stabilization. The area-based stomatal conductance (*g*_s_), transpiration rate (*E*), and the intercellular CO_2_ concentration (*C*_*i*_) were recorded by the Li-6400XT system simultaneously with the photosynthesis measurements.

### Leaf structure and biochemistry

Following the gas-exchange measurements, the leaves used for the measurement were harvested and brought to the laboratory in a cooling box. A leaf disc with an area of 2.14 cm^2^ was cut with a cork borer from each sampled leaf, freeze-dried for 24 h, and weighed. Subsequently, the discs were ground in a ball mill (Mixer Mill MM400; Retsch, Haan, Germany). Subsamples of 5–10 mg dry mass were extracted with 8 mL of 96% ethanol in the dark at room temperature for 24 h for pigment quantification. The concentrations of Chl *a*, Chl *b*, total Chl (*a* + *b*) and total carotenoid, and xanthophylls in the leaves were analyzed spectrophotometrically according to Lichtenthaler (1987). The concentration of total carbon (C) and nitrogen (N) in the leaves were analyzed by a CN Analyser (model NA2000; Fisons Instruments, Carlo Erba, Italy). The leaf ash contents (Ash) were determined by burning of dry leaf powder samples in a 450°C muffle furnace for 6 h. The heat of combustion (HC) was measured in approximately 200 mg of leaf powder using a Parr bomb calorimeter (model 6725; Parr Instrument Co., Moline, IL), calibrated with benzoic acid pellets with known energy contents. For both the Ash and HC measurements, triplicate samples were analyzed and averaged for each genotype.

### Leaf resource/energy-use efficiency

The intrinsic water use efficiency (IWUE) and the photosynthetic nitrogen use efficiency (PNUE) were calculated as the area-based *A* divided by *g*_*s*_ and the area-based N concentration, respectively (Jiang et al. 2009). The leaf respiration efficiency (RE) was obtained by dividing *A* by the corresponding *R*. A high RE indicates a low respiration cost for photosynthesis, hence allowing more carbon to be allocated to growth (Feng et al. 2007b, 2011).

The construction costs (CC), that is, the amount of glucose needed to form one g of leaf (Williams et al. 1987), was calculated using a formula based on the growth efficiency of the leaf tissue, the heat of combustion and the ash and nitrogen content (Williams et al. 1987):


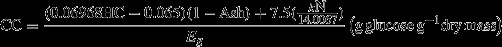
(2)

where HC is the ash-free heat of combustion (kJ·g^−1^); Ash is the ash content (g·g^−1^ leaf dry mass); N is the total nitrogen concentration (g·g^−1^ dry mass); *k* is the oxidation state of the nitrogen source (+5 for nitrate, −3 for ammonium); and *E*_g_ is the growth efficiency (the fraction of the energy required to provide a reductant that is consumed during the formation of the tissue, but is not incorporated into the biomass; Williams et al. 1987; Poorter et al. 2006). The value of *E*_g_ used in this study was 0.87 (Williams et al. 1987; Griffin 1994). It was assumed that the nitrogen source for all specimens was both nitrate and ammonium; CC was therefore calculated using both *k* = +5 and −3, and the average values were used. To calculate the leaf CC per unit leaf area, the obtained mass-based CC values were divided by the SLA (CC_area_, g·glucose·m^−2^).

The leaf photosynthetic energy-use efficiency (PEUE) was calculated as the ratio of *A*_area_ to CC_area_ (Feng et al. 2011). The payback time, that is, the time needed to recover the carbon invested in the construction of a leaf through photosynthesis (Poorter et al. 2006; Karagatzides and Ellison 2009), was calculated as CC_mass_/*A*_mass_ after converting CC_mass_ from g·glucose·g^−1^ dry mass to nmol·g^−1^ dry mass and *A*_mass_ from *μ*mol CO_2_ g^−1^ dry mass·s^−1^ to nmol C·g^−1^ dry mass·h^−1^ (Karagatzides and Ellison 2009).

### Data analysis

To assess differences between the three groups, we converted the mass-based parameters (e.g., N, C, chlorophyll) into area-based parameters *via* division by the corresponding SLA, while the area-based variables measured with the LI-COR system (e.g., *A*, *R*) were converted into mass-based variables through multiplication by the corresponding SLA.

Prior to the statistical analysis, all ecophysiological variables were log_10_ (value +1) transformed to satisfy the requirement of a normal distribution and homogeneity of variances, which were tested with the Shapiro–Wilk test and Levene's test, respectively. However, for clarity the untransformed data are presented. One-way analysis of variance (ANOVA) using the Type III sum of squares was used to compare the means between the three groups (AM, EU, and AMn). When the ANOVA results were significant, post hoc multiple comparisons of means were applied using Bonferroni's post hoc analysis (Statgraphics XV centurion v. 16.1.11; StatPoint, Inc. Warrenton, VA).

The standardized major axis (SMA) regression analysis of the SMATR software (Falster et al. 2006) was used to compare the bivariate allometric relationships between pairs of traits to identify differences in the correlations between ecophysiological traits, that is, possible different trait trade-offs, among groups. Superior to ANCOVA, SMA regression minimizes the residual variance in both the *x* and *y* dimensions (Warton and Weber 2002; Warton et al. 2006), and the statistic used in the SMA (WALD test) is independent of differences between groups in sample size, residual variances, and means of the *X* variable (Warton et al. 2006). The procedure for the SMA tests was threefold: First, the heterogeneity of the fitted slopes (S) among the different groups was evaluated. Next, when the slopes were homogeneous, a common slope for all groups was estimated *via* a likelihood ratio method. Finally, shifts in the SMA elevation (E) among the fitted slopes for each group and shifts along the common slope (CS) for each fitted slope were tested (Warton et al. 2006). We analyzed the pairwise relationships between the leaf biochemistry traits (Chl, C, N), leaf gas-exchange traits (*A*, *R*, *C*_*i*_, *E*, *g*_s_), leaf resource/energy-use efficiency traits (RE, PNUE, IWUE, CC, PEUE, payback time), and biomass allocation traits. Here, we only present the significant relationships among the leaf trait sets.

A discriminant analysis of principal components (DAPC; Jombart et al. 2010) was carried out to analyze dissimilarities between the AM, AMn, and EU groups for all of the significantly different traits detected previously by the ANOVAs. We used the −2 Log-likelihood ratio as the criterion for the DAPC. Wilks' *λ* test was applied to test the significance of the canonical axes from the DAPC using the JMP statistical software (v. 10; SAS Institute Inc., Cary, NC).

## Results

### Differences detected by the ANOVA

Of the 47 plant traits investigated, 20 differed significantly between the three groups of *P. australis* (Table 2). Most differences were observed between the two lineages or haplotypes, that is, haplotype M (EU and AM) and haplotype E (AMn). Plants of the EU group differed significantly from plants of the AMn group in 10 plant traits, whereas plants of the AM group differed in a total of 15 traits from plants of the AMn group. Six of these traits were the same (mainly gas-exchange traits), but four were unique for the EU group (higher leaf N concentration, lower C:N-ratio, higher leaf dry matter content, and smaller basal stem diameter than the AMn group), and nine were unique for the AM group (higher *A*_area_ and *A*_mass_, higher PNUE and PEUE, lower payback time, higher Chl *b* content, more biomass allocation to leaf sheaths, and less allocation to stems than the AMn group). Within the haplotype M lineage, the introduced AM group differed from its ancestral native EU group in only two traits, as the AM group had significantly higher stem diameters and allocated more biomass to the leaf sheath than the EU group.

**Table 2:** Significantly different ecophysiological traits among the three groups of *Phragmites australis* (EU: native European; AM: introduced North American; AMn: native North American) and one-way ANOVA *F*-ratios.

	EU (*n* = 6)	AM (*n* = 9)	AMn (*n* = 5)	*F*-ratio
*A*_area_ (*μ*mol CO_2_ m^−2^·s^−1^)	18.1 (1.4)ab	20.1 (0.5)b	15.4 (0.8)a	5.86*
*A*_mass_ (*μ*mol CO_2_ g^−1^·s^−1^)	0.22 (0.01)ab	0.23 (0.01)b	0.18 (0.01)a	6.96**
*g*_s_ (mol H_2_O m^−2^·s^−1^)	0.28 (0.03)b	0.34 (0.01)b	0.19 (0.02)a	11.46**
*E* (mmol H_2_O m^−2^·s^−1^)	4.4 (0.3)b	4.9 (0.1)b	3.5 (0.2)a	10.94**
*C*_*i*_ (*μ*mol CO_2_ mol^−1^)	244 (4)b	251 (3)b	225 (5)a	11.90**
Leaf N content (mg·g^−1^)	2.62 (0.11)b	2.50 (0.04)ab	2.22 (0.15)a	4.20*
C:N-ratio	17.7 (0.7)a	18.3 (0.4)ab	20.7 (1.3)b	4.06*
PNUE (*μ*mol CO_2_ g^−1^ N·s^−1^)	8.33 (0.43)ab	9.38 (0.24)b	8.22 (0.22)a	4.82*
IWUE (*μ*mol CO_2_ mmol^−1^ H_2_O)	68 (3)a	62 (2)a	83 (4)b	13.13***
CC_mass_ (g glucose·g^−1^ dry mass)	1.41 (0.01)b	1.39 (0.01)b	1.36 (0.01)a	13.55***
PEUE (*μ*mol CO_2_ g^−1^·glucose·s^−1^)	0.155 (0.010)ab	0.167 (0.004)b	0.133 (0.007)a	5.88*
Payback time (h)	448 (29)ab	407 (10)a	516 (29)b	6.25**
Chl *b* (mg·g^−1^ dry mass)	1.07 (0.05)ab	1.09 (0.03)b	0.86 (0.09)a	5.31*
Chl *b* (mg·m^−2^)	90 (6)ab	94 (2)b	73 (7)a	4.80*
Leaf thickness (*μ*m)	19.4 (0.6)	19.4 (0.4)	21.8 (0.9)	4.14*
LDMC (mg·g^−1^)	368 (6)a	343 (8)ab	327 (10)b	5.02*
Basal stem diameter (mm)	5.4 (0.3)a	6.3 (0.2)b	7.4 (0.3)b	10.82**
Shoot dry matter content (mg·g^−1^)	284 (4)a	273 (5)a	232 (8)b	19.89***
P_Leaf sheath_ (%)	23.1 (0.7)a	26.8 (0.7)b	22.6 (1.9)a	13.59***
P_Stem_ (%)	45.0 (1.5)ab	41.7 (1.2)a	48.6 (1.8)b	5.65*

Values are means (SE). Different letters in the same row indicate significant differences among groups from Bonferroni's post hoc analysis at the 95.0% confidence level. *A*, leaf light-saturated photosynthetic assimilation; *g*_s_, leaf stomatal conductance; *E*, leaf transpiration rate; *C*_*i*_, leaf intercellular CO_2_ concentration; PNUE, leaf photosynthetic nitrogen use efficiency; IWUE, leaf intrinsic water use efficiency; CC_mass_, mass-based leaf construction cost; PEUE, leaf photosynthetic energy-use efficiency; LDMC, leaf dry matter content; P_leaf sheath_ and P_stem_ are proportions of leaf sheath and stem biomass to the total shoot biomass, respectively.

* *P* < 0.05,

** *P* < 0.01,

*** ***P < 0.001.

### Differences detected by the standardized major axis (SMA) regressions

**Figure 1:**
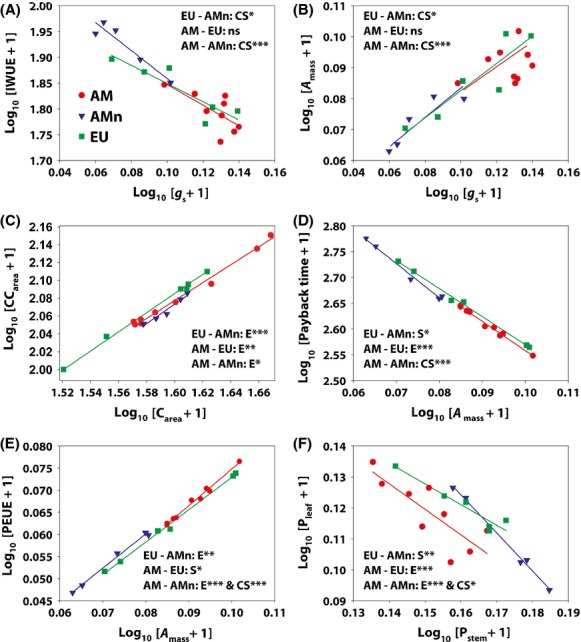
Selected significantly different pairwise relationships in standardized major axis (SMA) analysis between biochemical, gas exchange, resource/energy-use efficiency, and biomass allocation traits of EU (native European), AM (introduced North American), and AMn (native North American). The complete results of SMA analysis and statistical significance of the relationships (*r*^2^ and *P*-value) are shown in Tables S2–S4. *g*_s_: leaf stomatal conductance; IWUE: leaf intrinsic water use efficiency; *A*_mass_, mass-based leaf light-saturated photosynthetic capacity; C_area_: area-based leaf carbon content; CC_area_: area-based leaf construction cost; PEUE: leaf photosynthetic energy-use efficiency; P_stem_ and P_leaf_ are proportions of stem and leaf biomass to the total shoot biomass, respectively. The three regression lines were tested for differences in slope (S), elevation (E), and shift along the common fitted slope (CS) (ns, p > 0.05; *P < 0.05; **P < 0.01; *** *P* < 0.001).

The SMA tests found 25 significantly different pairs of traits for the EU-AMn comparisons, 20 pairs for the AM-EU comparisons, and 81 pairs for AM-AMn comparisons (Tables S2–S4). Selected relationships with significantly regression lines are shown in Figure 1, and the complete statistics of the SMA regression analyses are presented in Tables S2–S4.

For the *g*_s_–IWUE relationship (Fig. 1A) and the *g*_s_–*A*_mass_ relationship (Fig. 1B), it is seen that there was no significant difference between the AM and EU groups, but a significant difference between the EU and AMn groups, and an even more significant difference between the AM and AMn groups. These differences were caused by the fact that the *g*_s_ of the AMn group consistently were lower (located to the left in the plots) than the *g*_s_ of the AM group (points located to the right in the plots) whereas the range of *g*_s_ of the EU group was larger and overlapping with both the ranges for the AM and the AMn group. It is also evident from the plots that significant shifts in the elevation (E) of the fitted lines were observed for all pairs of the three groups for the C_area_–CC_area_ relationship (Fig. 1C), with the AMn group having the lowest and the EU group the highest construction cost at a given leaf carbon content per unit leaf area, and with the AM group resembling the AMn group more than the EU group. For the relationship between *A*_mass_ and payback time (Fig. 1D), the EU and AMn groups had heterogeneous slopes (S), the AM and the EU groups differed in the elevation (E) of the fitted line, and the AM and AMn groups shifted along the common SMA slope (CS). For the *A*_mass_–PEUE relationship (Fig. 1E), the AMn group also had different relationships with the EU and AM groups as the photosynthetic energy-use efficiency (PEUE) consistently was higher for a given *A*_mass_ for the AM and AMn groups compared to the EU group. Also, the PEUE of the AM group increased more with *A*_mass_ than the EU group, as indicated by the significant difference in the slope (S) of the regression lines for the two groups (Fig. 1E). For the relationship between biomass allocation of leaf and stem (Fig. 1F), the EU group differed in slope (S) from the AMn group, but not the AM group, while the AM group in general allocated less biomass to leaves at a given stem biomass than the EU and AMn groups as indicated by the shifts in elevation (E) and had lower stem biomass relative to the AMn group (CS).

### Results of the multivariate tests

**Figure 2:**
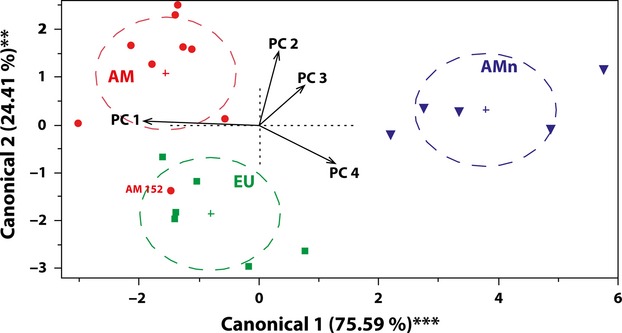
Discriminant analysis of principal components (DAPC) ordination for the three groups using all significantly different variables (Tables 2 and S5). EU: native European; AM: introduced North American; AMn: native North American. The directions and lengths (×1.5) of the Principal Component axis (black arrows) represent the correlation with the first two canonical axes. The dashed circles and crosses are 95% confidence ellipses, and centroids for each group, respectively. The significance of the discriminant functions was tested by Wilks' *λ* test, **P < 0.01, ***P < 0.001.

The discriminant analysis of principal components (DAPC) of the significantly different traits detected by the ANOVAs identified four significant PC axes that explained 85.9% of the total variation (Table S5). Using these four axes as the input variables, the DAPC separated the haplotype E lineage (the AMn group) as being significantly different from the haplotype M lineage (the EU and AM groups) along the first canonical axis (Fig. 2, Wilks' *λ* = 0.05, *P* < 0.001, 75.3% of variation explained). This axis had the highest factor loadings for gas-exchange parameters (*A*, *g*_s_, *E*, *C*_*i*_), photosynthetic energy-use efficiency (PEUE), and payback time (all contributing with loadings higher than 80% to axis 1; Table S5). The DAPC analysis did not resolve the AM and EU groups completely, as one AM genotype (AM 152) clustered in the EU group with a high probability (>93%, Table S6 and Fig. 2). However, the analysis significantly separated the EU group from the AM group along the second canonical axis (Wilks' *λ* = 0.35, *P* < 0.01, 24.7% of variation explained), which had high loadings for photosynthetic nitrogen use efficiency (PNUE; 59% of variation explained) and construction costs (CC_mass_; 46% of variation explained).

## Discussion

We here present some of the first data indicating, that the Eurasian invasive haplotype M *P. australis* was preadapted to become invasive prior to its introduction in North America, and moreover that several invasive traits evolved in the new range after its introduction. It might be argued, that our sampling scheme in the native range missed sampling the native EU populations that gave rise to the invasive haplotype. However, the EU genotypes selected originated from populations widely dispersed throughout the native-range distribution area in Europe, and likely represented highly competitive genotypes. Correspondingly, we also found that the niche space of the introduced population along the East Coast of North America differed from that of the European native-range population (Guo et al. 2013), and the invasive population is genetically differentiated from the European one (Lambertini et al. 2012b). We are therefore confident, that our sampling scheme includes a representative sample set of the population studied over their distribution ranges, but a larger set of genotypes from each population would of course have made our findings and conclusions stronger.

The trait comparisons performed in the common environment allowed us to distinguish traits possessed in Europe before the introduction to North America (i.e., traits shared by the native range [EU] and introduced [AM] populations of the invasive lineage, but different from the traits of the native North American conspecific population [AMn]) and traits evolved during the invasion process (i.e., traits diverging between the native range [EU] and the introduced [AM] groups). The results indicate that the invasive AM inherited from its ancestor EU a more efficient photosynthetic apparatus than that of the native North American conspecific *P. australis* ssp. *americanus* (AMn). The competitive advantage of the invasive AM group in photosynthetic capacity over the native conspecific AMn is therefore an advantageous trait acquired before the introduction, that is, AM was preadapted to become invasive in North America. Mozdzer and Zieman (2010) and Mozdzer and Megonigal (2012) found similar differences in *A*_max_ and other photosynthesis-related traits between genotypes of invasive AM and native AMn groups in experiments conducted in North America using local genotypes from the Atlantic Coast.

The high photosynthetic assimilation rates of the introduced AM affected several photosynthesis-related traits. The traits diverged slightly, but significantly, among the native range EU and the introduced AM in the direction of a more advantageous payback time and photosynthetic energy-use efficiency (PEUE). Evidence of evolutionary change in the ecophysiological traits investigated was further confirmed by the DAPC, which separated the introduced AM group from the native range EU group when all traits were analyzed together (Fig. 2). The SMA analysis also showed differences in the ranges of *A*_mass_ values among the groups. The range of *A*_mass_ for the introduced AM fell in the upper range of that of the native range EU group. This difference might result from the evolutionary processes in the introduced range after introduction due to genetic selection and/or founder effect, but also by changes in the native range in Europe. In our study, not all AM genotypes showed the same degree of differentiation from the native range EU genotypes, as one of the AM genotypes (AM 152) appeared to have retained the European traits in the DAPC analysis. This can, however, be expected both for traits under selection, and in the case of multiple asynchronous introductions from Europe (Hauber et al. 2011; Plut et al. 2011; Lambertini et al. 2012b; Meyerson and Cronin 2013) because of founder effect and/or genetic drift. Further research of the ecophysiological variation patterns in relation to the genetic diversity patterns in North America and Europe is necessary to understand the evolutionary implications of the differences observed in both ranges.

In addition to the differences detected in the photosynthesis-related traits, we also found divergent morphological traits. The stem diameter of haplotype M *P. australis* was higher in the introduced range than in the native range, and the allocation of biomass to leaf sheaths was higher for invasive AM than for the European native range EU population. In the Poaceae, the leaf sheath provides physical support to stems and leaves, protects the intermediate nodal meristems, axillary buds and vascular tissues, acts as a channel to transport nutrients and photosynthetic products (Haslam 1972; Liu et al. 2011) and enhances the transport of oxygen to the belowground parts of the plant *via* a pressurized gas through-flow mechanism (Konnerup et al. 2011). Hence, a thicker stem and more robust leaf sheath could provide advantages to the plants.

Contrary to the findings of Mozdzer and Zieman (2010) and Mozdzer and Megonigal (2012), we did not observe any significant differences in SLA between the AM and the AMn group, and not even between the EU and the AMn group, despite the fact that the SLA was analyzed twice at different times during the growing season and using two different commonly applied methods (Table S1). Although invasive plants generally exhibit a greater SLA, some studies have observed similar or even lower SLA values for invasive species than non-invasive congeners (e.g., Nagel and Griffin 2001; McDowell 2002; Feng et al. 2007a).

## Conclusion

Based on the ecophysiological and morphological traits of today's populations, we suggest that the European lineage of the wetland grass *P. australis* was preadapted to be invasive in North America prior to its introduction, as it possesses several superior ecophysiological traits in comparison to the native North American conspecific *P. australis* ssp. *americanus*. We also provide evidence that several invasive traits have evolved as the introduction more than two centuries ago and have differentiated the introduced population from its relatives in Europe. As such invasive traits confer a competitive advantage over the native North American *P. australis* ssp. *americanus*, we suggest that the innate invasiveness of the European lineage evolved further after the introduction. Our approach has the advantage of disentangling genetically determined variation from acclimation and phenotypic plasticity, but does not address the role of the environment in the evolution of the traits analyzed. Further research is therefore needed to link the observed changes to the genetic variation and the interaction with the environment and shed conclusively light on the evolutionary processes that have been occurring in North America and Europe.

## Note

1 Traits are defined as any morphological, physiological, or phenological traits that are measurable at the individual level and that impact fitness indirectly *via* their effects on growth, reproduction, and survival (Violle et al. 2007).

## Supporting Information

**Table S1.** Traits analysed in the study which were not statically significantly different in the one-way analysis of variance (ANOVA) between the groups (EU: native European; AM: introduced North American; AMn: native North American). **Table S2.** Results of standardized major axis (SMA) regression analysis between EU and AMn. **Table S3.** Results of standardized major axis (SMA) regression analysis between AM and EU. **Table S4.** Results of standardized major axis (SMA) regression analysis between AM and AMn. **Table S5.** Factor loadings in PCA. **Table S6.** Discriminant analysis for genotypes based on the groups of origins (EU: native European; AM: introduced North American; AMn: native North American).

## References

[b1] Alexander JM, van Kleunen M, Ghezzi R, Edwards PJ (2012). Different genetic clines in response to temperature across the native and introduced ranges of a global plant invader. J. Ecol.

[b2] Atwood J, Meyerson L (2011). Beyond EICA: understanding post-establishment evolution requires a broader evaluation of potential selection pressures. NeoBiota.

[b3] Barrett SCH, Colautti RI, Eckert CG (2008). Plant reproductive systems and evolution during biological invasion. Mol. Ecol.

[b4] Bennett AE, Thomsen M, Strauss SY (2011). Multiple mechanisms enable invasive species to suppress native species. Am. J. Bot.

[b5] Bossdorf O, Auge H, Lafuma L, Rogers WE, Siemann E, Prati D (2005). Phenotypic and genetic differentiation between native and introduced plant populations. Oecologia.

[b6] Bucharova A, van Kleunen M (2009). Introduction history and species characteristics partly explain naturalization success of North American woody species in Europe. J. Ecol.

[b7] Clevering OA, Lissner J (1999). Taxonomy, chromosome numbers, clonal diversity and population dynamics of *Phragmites australis*. Aquat. Bot.

[b8] Falster DS, Warton DI, Wright IJ (2006). http://bio.mq.edu.au/research/groups/ecology//SMATR/index.html.

[b9] Feng Y-L, Wang J, Sang W (2007a). Biomass allocation, morphology and photosynthesis of invasive and noninvasive exotic species grown at four irradiance levels. Acta Oecol.

[b10] Feng Y-L, Auge H, Ebeling SK (2007b). Invasive *Buddleja davidii* allocates more nitrogen to its photosynthetic machinery than five native woody species. Oecologia.

[b11] Feng Y-L, Li Y-P, Wang R-F, Callaway RM, Valiente-Banuet A, Inderjit (2011). A quicker return energy-use strategy by populations of a subtropical invader in the non-native range: a potential mechanism for the evolution of increased competitive ability. J. Ecol.

[b12] Griffin KL (1994). Calorimetric estimates of construction cost and their use in ecological studies. Funct. Ecol.

[b13] Guo WY, Lambertini C, Li XZ, Meyerson LA, Brix H (2013). Invasion of Old World *Phragmites australis* in the New World: precipitation and temperature patterns combined with human influences redesign the invasive niche. Glob. Change Biol.

[b14] Gurevitch J, Fox GA, Wardle GM, Inderjit, Taub D (2011). Emergent insights from the synthesis of conceptual frameworks for biological invasions. Ecol. Lett.

[b15] Haslam SM (1972). *Phragmites communis* Trin. (*Arundo phragmites* L.,? *Phragmites australis* (Cav.) Trin. ex Steudel). J. Ecol.

[b16] Hauber DP, Saltonstall K, White DA, Hood CS (2011). Genetic variation in the common reed, *Phragmites australis*, in the Mississippi River Delta Marshes: evidence for multiple introductions. Estuaries Coasts.

[b17] Heberling JM, Fridley JD (2013). Resource-use strategies of native and invasive plants in Eastern North American forests. New Phytol.

[b18] Henery ML, Bowman G, Mraz P, Treier UA, Gex-Fabry E, Schaffner U (2010). Evidence for a combination of pre-adapted traits and rapid adaptive change in the invasive plant Centaurea stoebe. J. Ecol.

[b19] Hierro JL, Maron JL, Callaway RM (2005). A biogeographical approach to plant invasions: the importance of studying exotics in their introduced and native range. J. Ecol.

[b20] Jiang L, Luo Y, Chen J, Li B (2009). Ecophysiological characteristics of invasive *Spartina alterniflora* and native species in salt marshes of Yangtze River estuary, China. Estuar. Coast. Shelf Sci.

[b21] Jombart T, Devillard S, Balloux F (2010). Discriminant analysis of principal components: a new method for the analysis of genetically structured populations. BMC Genet.

[b22] Karagatzides JD, Ellison AM (2009). Construction costs, payback times, and the leaf economics of carnivorous plants. Am. J. Bot.

[b23] Keller SR, Taylor DR (2008). History, chance and adaptation during biological invasion: separating stochastic phenotypic evolution from response to selection. Ecol. Lett.

[b24] van Kleunen M, Dawson W, Schlaepfer D, Jeschke JM, Fischer M (2010a). Are invaders different? A conceptual framework of comparative approaches for assessing determinants of invasiveness. Ecol. Lett.

[b25] van Kleunen M, Weber E, Fischer M (2010b). A meta-analysis of trait differences between invasive and non-invasive plant species. Ecol. Lett.

[b26] van Kleunen M, Schlaepfer DR, Glaettli M, Fischer M (2011). Preadapted for invasiveness: do species traits or their plastic response to shading differ between invasive and non-invasive plant species in their native range?. J. Biogeogr.

[b27] Konnerup D, Sorrell BK, Brix H (2011). Do tropical wetland plants possess convective gas flow mechanisms?. New Phytol.

[b28] Kumschick S, Hufbauer RA, Alba C, Blumenthal DM (2013). Evolution of fast-growing and more resistant phenotypes in introduced common mullein (*Verbascum thapsus*. J. Ecol.

[b29] Lambertini C, Gustafsson MHG, Frydenberg J, Lissner J, Speranza M, Brix H (2006). A phylogeographic study of the cosmopolitan genus *Phragmites* (Poaceae) based on AFLPs. Plant Syst. Evol.

[b30] Lambertini C, Mendelssohn IA, Gustafsson MH, Olesen B, Riis T, Sorrell BK (2012a). Tracing the origin of Gulf Coast *Phragmites* (Poaceae): a story of long-distance dispersal and hybridization. Am. J. Bot.

[b31] Lambertini C, Sorrell BK, Riis T, Olesen B, Brix H (2012b). Exploring the borders of European *Phragmites* within a cosmopolitan genus. AoB Plants.

[b32] Larkin DJ, Freyman MJ, Lishawa SC, Geddes P, Tuchman NC (2011). Mechanisms of dominance by the invasive hybrid cattail *Typha* × *glauca*. Biol. Invasions.

[b33] Leishman MR, Haslehurst T, Ares A, Baruch Z (2007). Leaf trait relationships of native and invasive plants: community- and global-scale comparisons. New Phytol.

[b34] Li F, Yang Q, Zan Q, Tam NF, Shin PK, Vrijmoed LL (2011). Differences in leaf construction cost between alien and native mangrove species in Futian, Shenzhen, China: implications for invasiveness of alien species. Mar. Pollut. Bull.

[b35] Lichtenthaler HK (1987). Chlorophylls and carotenoids: pigments of photosynthetic biomembranes. Methods Enzymol.

[b36] Liu H, Rao Y, Yang Y, Leng Y, Huang L, Zhang G (2011). Genetic analysis of traits related to leaf sheath in rice (*Oryza sativ*a L.). Mol. Plant Breed.

[b37] McDowell SCL (2002). Photosynthetic characteristics of invasive and noninvasive species of *Rubus* (Rosaceae). Am. J. Bot.

[b38] Meyerson LA, Cronin JT (2013). Evidence for multiple introductions of *Phragmites australis* to North America: detection of a new non-native haplotype. Biol. Invasions.

[b39] Meyerson LA, Saltonstall K, Chambers RM, Bertness MD, Silliman BR, Grosholz E (2009). *Phragmites australis* in eastern North America: a historical and ecological perspective. Salt marshes under global Siege.

[b40] Meyerson LA, Lambertini C, McCormick MK, Whigham DF (2012). Hybridization of common reed in North America? The answer is blowing in the wind. AoB Plants.

[b41] Mozdzer TJ, Megonigal JP (2012). Jack-and-master trait responses to elevated CO_2_ and N: a comparison of native and introduced *Phragmites australis*. PLoS ONE.

[b42] Mozdzer TJ, Zieman JC (2010). Ecophysiological differences between genetic lineages facilitate the invasion of non-native *Phragmites australis* in North American Atlantic coast wetlands. J. Ecol.

[b43] Nagel JM, Griffin KL (2001). Construction cost and invasive potential: comparing *Lythrum salicaria* (Lythraceae) with co-occurring native species along pond banks. Am. J. Bot.

[b44] Nentwig W (2007). Biological invasions.

[b45] Osunkoya OO, Bayliss D, Panetta FD, Vivian-Smith G (2010). Leaf trait co-ordination in relation to construction cost, carbon gain and resource-use efficiency in exotic invasive and native woody vine species. Ann. Bot.

[b46] Parker JD, Torchin ME, Hufbauer RA, Lemoine NP, Alba C, Blumenthal DM (2013). Do invasive species perform better in their new ranges?. Ecology.

[b47] Plut K, Paul J, Ciotir C, Major M, Freeland JR (2011). Origin of non-native *Phragmites australis* in North America, a common wetland invader. Fundam. Appl. Limnol.

[b48] Poorter H, Pepin S, Rijkers T, de Jong Y, Evans JR, Korner C (2006). Construction costs, chemical composition and payback time of high- and low-irradiance leaves. J. Exp. Bot.

[b49] Pyšek P, W Nentwig, Richardson D (2007). Traits associated with invasiveness in alien plants: where do we stand?. Biological invasions.

[b50] Pyšek P, Jarošík V, Pergl J, Randall R, Chytrý M, Kühn I (2009). The global invasion success of Central European plants is related to distribution characteristics in their native range and species traits. Divers. Distrib.

[b51] Qin R-M, Zheng Y-L, Valiente-Banuet A, Callaway RM, Barclay GF, Pereyra CS (2013). The evolution of increased competitive ability, innate competitive advantages, and novel biochemical weapons act in concert for a tropical invader. New Phytol.

[b52] Richardson DM, Pyšek P (2006). Plant invasions: merging the concepts of species invasiveness and community invasibility. Prog. Phys. Geogr.

[b53] Riis T, Lambertini C, Olesen B, Clayton JS, Brix H, Sorrell BK (2010). Invasion strategies in clonal aquatic plants: are phenotypic differences caused by phenotypic plasticity or local adaptation?. Ann. Bot.

[b54] Saltonstall K (2002). Cryptic invasion by a non-native genotype of the common reed, *Phragmites australis*, into North America. Proc. Natl Acad. Sci. U.S.A.

[b55] Schlaepfer DR, Glattli M, Fischer M, van Kleunen M (2010). A multi-species experiment in their native range indicates pre-adaptation of invasive alien plant species. New Phytol.

[b56] Vile D, Garnier E, Shipley B, Laurent G, Navas ML, Roumet C (2005). Specific leaf area and dry matter content estimate thickness in laminar leaves. Ann. Bot.

[b57] Violle C, Navas M-L, Vile D, Kazakou E, Fortunel C, Hummel I (2007). Let the concept of trait be functional!. Oikos.

[b58] Warton D, Weber N (2002). Common slope tests for bivariate structural relationships. Biomet. J.

[b59] Warton DI, Wright IJ, Falster DS, Westoby M (2006). Bivariate line-fitting methods for allometry. Biol. Rev.

[b60] Whitney KD, Gabler CA (2008). Rapid evolution in introduced species, ‘invasive traits’ and recipient communities: challenges for predicting invasive potential. Divers. Distrib.

[b61] Williams K, Percival F, Merino J, Mooney HA (1987). Estimation of tissue construction cost from heat of combustion and organic nitrogen content. Plant, Cell Environ.

[b62] Zedler JB, Kercher S (2004). Causes and consequences of invasive plants in wetlands: opportunities, opportunists, and outcomes. Crit. Rev. Plant Sci.

[b63] Zou J, Rogers WE, Siemann E (2007). Differences in morphological and physiological traits between native and invasive populations of *Sapium sebiferum*. Funct. Ecol.

