# Supplementary material for: Preadaptation and post-introduction evolution facilitate the invasion of Phragmites australis in North America
Source: Ecol Evol. 2014 Nov 25;4(24):4567–77. doi: 10.1002/ece3.1286 (PMC4278810; doi:10.1002/ece3.1286)
Supplement: Supplementary file 1 [file ece30004-4567-sd1.docx]

**Table S1**. Traits analysed in the study which were not statically significantly different in the one-way analysis of variance (ANOVA) between the groups (EU: native European; AM: introduced North American; AMn: native North American.). Values are means (S.E.). Leaf shape: ratio of leaf width to leaf length; SLA: specific leaf area; P_leaf_ is the proportion of leaf biomass to the total shoot biomass; *R*: leaf respiration; RE: leaf respiration efficiency; HC: heat of combustion; Ash: leaf ash content; CC_area_: area-based leaf construction cost.

|  | **EU**  (n = 6) | **AM**  (n = 9) | **AMn**  (n = 5) | **F-ratio** |
| --- | --- | --- | --- | --- |
| Leaf area (cm^2^) | 59.2 (5.0) | 68.6 (3.4) | 56.2 (3.6) | 2.70 |
| Leaf width (mm) | 28.8 (1.6) | 30.5 (1.0) | 29.2 (1.3) | 0.61 |
| Leaf length (mm) | 370 (7) | 388 (12) | 349 (12) | 2.72 |
| Leaf shape | 0.078 (0.003) | 0.079 (0.002) | 0.084 (0.003) | 0.48 |
| Leaf dry mass (g) | 0.42 (0.04) | 0.46 (0.03) | 0.40 (0.04) | 0.83 |
| SLA_May_ (m^2^ kg^-1^ dry mass) | 141 (3) | 151 (6) | 142 (8) | 0.75 |
| Shoot height (mm) | 1337 (41) | 1357 (40) | 1385 (58) | 0.20 |
| Shoot dry mass (g) | 5.92 (0.44) | 7.05 (0.57) | 7.02 (0.74) | 1.12 |
| SLA_July_ (m^2^ kg^-1^ dry mass) | 12.2 (0.5) | 11.7 (0.3) | 11.8 (0.2) | 0.47 |
| P_Leaf_ (%) | 31.9 (1.0) | 31.4 (1.1) | 28.8 (1.9) | 1.41 |
| Chl *a* _mass_ (mg g^-1^ dry mass) | 0.28 (0.02) | 0.28 (0.01) | 0.23 (0.02) | 2.94 |
| Chl *a* _area_ (g m^-2^) | 3.32 (0.19) | 3.29 (0.10) | 2.77 (0.30) | 3.10 |
| Chl *a*/*b* | 3.11 (0.08) | 3.03 (0.08) | 3.22 (0.02) | 1.70 |
| Chl *a*+*b* _mass_ (mg g^-1^ dry mass) | 0.37 (0.03) | 0.38 (0.01) | 0.31 (0.03) | 3.68 |
| Chl *a*+*b* _area_(g m^-2^) | 4.39 (0.24) | 4.38 (0.13) | 3.63 (0.39) | 3.10 |
| Carotenoids _mass_ (mg g^-1^ dry mass) | 0.065 (0.005) | 0.062 (0.003) | 0.060 (0.003) | 0.49 |
| Carotenoids _area_ (g m^-2^) | 0.79 (0.06) | 0.73 (0.04) | 0.70 (0.04) | 0.62 |
| Chl (*a*+*b*)/Carotenoids | 5.71 (0.39) | 6.16 (0.30) | 5.12 (0.25) | 2.72 |
| C_mass_ (% dry mass) | 45.3 (0.2) | 45.4 (0.1) | 45.0 (0.2) | 1.11 |
| C_area_ (g m^-2^) | 37.7 (1.5) | 39.4 (1.2) | 38.6 (0.5) | 0.49 |
| N_area_ (g m^-2^) | 2.19 (0.14) | 2.16 (0.05) | 1.89 (0.12) | 2.33 |
| *R*_area_ (μmol CO_2_ m^-2^ s^-1^) | 2.54 (0.18) | 2.28 (0.11) | 2.32 (0.27) | 0.64 |
| *R*_mass_ (μmol CO_2_ g^-1^ s^-1^) | 0.030 (0.002) | 0.026 (0.001) | 0.027 (0.003) | 1.37 |
| RE | 8.15 (1.24) | 9.75 (0.57) | 7.05 (0.35) | 2.99 |
| HC (KJ g^-1^) | 18.08 (1.06) | 18.91 (0.04) | 18.65 (0.11) | 0.70 |
| Ash (g g^-1^) | 0.044 (0.001) | 0.044 (0.002) | 0.048 (0.002) | 1.66 |
| CC_area_ (g glucose m^-2^) | 117 (5) | 120 (4) | 116 (2) | 0.34 |

Table S2 Results of standardized major axis (SMA) regression analysis between EU and AMn. Significant results (P < 0.05) are shown in bold. Traits marked in red colour indicate relationships plotted in Fig.1. C_mass_ and C_area_: mass-and area based leaf carbon concentration; N_mass_ and N_area_: mass-and area based leaf nitrogen concentration; *A*_mass_ and *A*_area_: mass-and area based leaf light-saturated photosynthetic assimilation; R_area_: area based leaf respiration; *g*_s_: stomatal conductance; *C_i_*: intercellular CO_2_ concentration; IWUE: leaf intrinsic water use efficiency; CC_mass_: mass-based leaf construction cost; RE: leaf respiration efficiency; PEUE: leaf photosynthetic energy-use efficiency; P_leaf sheath_, P_leaf_, and P_stem_ are proportions of leaf sheath, leaf, and stem biomass to the total shoot biomass, respectively.

| Trait X | Trait Y | | group | r^2^ | *p* | Slope | Intercept | Slope homogeneity (S) | Shift in elevation (E) | Shift along the common slope (CS) |
| --- | --- | --- | --- | --- | --- | --- | --- | --- | --- | --- |
| C_mass_ | | IWUE | EU  AMn | 0.356  0.000 | 0.211  0.993 | 10.44  12.31 | -15.55  -18.55 | 0.842 | **0.007** | 0.272 |
|  |  |  | pooled | 0.008 | 0.792 | 14.67 | -22.54 |  |  |  |
|  |  | CC_mass_ | EU  AMn | 0.805  0.501 | 0.015  0.181 | 0.59  0.70 | -0.599  -0.791 | 0.740 | **0.000** | **0.007** |
|  |  |  | pooled | 0.407 | 0.035 | 1.157 | -1.547 |  |  |  |
| C_area_ | | IWUE | EU | 0.068 | 0.618 | -1.26 | 3.840 | 0.198 | **0.032** | 0.237 |
|  |  |  | AMn | 0.010 | 0.876 | -3.76 | 7.924 |  |  |  |
|  |  |  | pooled | 0.003 | 0.875 | -2.14 | 5.280 |  |  |  |
|  |  | CC_mass_ | EU | 0.322 | 0.241 | 0.07 | 0.269 | 0.151 | **0.000** | **0.023** |
|  |  |  | AMn | 0.078 | 0.649 | -0.21 | 0.715 |  |  |  |
|  |  |  | pooled | 0.005 | 0.837 | 0.17 | 0.110 |  |  |  |
|  |  | CC_area_ | EU | 0.996 | 0.000 | 1.06 | 0.384 | 0.715 | **0.000** | 0.883 |
|  |  |  | AMn | 0.948 | 0.005 | 1.12 | 0.279 |  |  |  |
|  |  |  | pooled | 0.958 | 0.000 | 1.06 | 0.388 |  |  |  |
| N_mass_ | | *A*_mass_ | EU | 0.433 | 0.155 | 0.40 | -0.138 | 0.127 | 0.760 | **0.0498** |
|  |  |  | AMn | 0.799 | 0.041 | 0.18 | -0.203 |  |  |  |
|  |  |  | pooled | 0.639 | 0.003 | 0.28 | -0.070 |  |  |  |
|  |  | IWUE | EU | 0.029 | 0.746 | -1.64 | 2.749 | 0.583 | 0.661 | **0.016** |
|  |  |  | AMn | 0.330 | 0.311 | -1.09 | 2.474 |  |  |  |
|  |  |  | pooled | 0.386 | 0.041 | -1.47 | 2.659 |  |  |  |
|  |  | CC_mass_ | EU | 0.508 | 0.112 | 0.09 | 0.331 | 0.589 | 0.112 | **0.000** |
|  |  |  | AMn | 0.021 | 0.814 | 0.06 | 0.342 |  |  |  |
|  |  |  | pooled | 0.463 | 0.021 | 0.12 | 0.317 |  |  |  |
| N_area_ | | IWUE | EU | 0.082 | 0.583 | -1.05 | 2.361 | 0.847 | 0.334 | 0**.029** |
|  |  |  | AMn | 0.392 | 0.259 | -1.21 | 2.479 |  |  |  |
|  |  |  | pooled | 0.322 | 0.064 | -1.35 | 2.526 |  |  |  |
|  |  | CC_mass_ | EU | 0.481 | 0.126 | 0.06 | 0.353 | 0.847 | **0.001** | **0.011** |
|  |  |  | AMn | 0.001 | 0.966 | 0.07 | 0.342 |  |  |  |
|  |  |  | pooled | 0.349 | 0.056 | 0.11 | 0.327 |  |  |  |
| *A*_area_ | | IWUE | EU | 0.475 | 0.130 | -0.60 | 2.600 | 0.425 | 0.183 | **0.045** |
|  |  |  | AMn | 0.553 | 0.150 | -0.96 | 3.085 |  |  |  |
|  |  |  | pooled | 0.552 | 0.009 | -0.86 | 2.946 |  |  |  |
|  |  | CC_mass_ | EU | 0.279 | 0.281 | 0.03 | 0.339 | 0.523 | **0.021** | **0.000** |
|  |  |  | AMn | 0.068 | 0.673 | -0.05 | 0.440 |  |  |  |
|  |  |  | pooled | 0.241 | 0.125 | 0.07 | 0.294 |  |  |  |
| *A*_mass_ | | IWUE | EU | 0.446 | 0.147 | -4.08 | 2.186 | 0.467 | 0.549 | **0.024** |
|  |  |  | AMn | 0.656 | 0.096 | -5.95 | 2.353 |  |  |  |
|  |  |  | pooled | 0.649 | 0.003 | -5.25 | 2.294 |  |  |  |
|  |  | CC_mass_ | EU | 0.128 | 0.486 | 0.23 | 0.362 | 0.641 | 0.089 | **0.000** |
|  |  |  | AMn | 0.020 | 0.820 | -0.34 | 0.398 |  |  |  |
|  |  |  | pooled | 0.309 | 0.076 | 0.41 | 0.345 |  |  |  |
|  |  | Payback time | EU | 0.997 | 0.000 | -5.44 | 3.113 | **0.044** | - | - |
|  |  |  | AMn | 0.989 | 0.001 | -6.69 | 3.195 |  |  |  |
|  |  |  | pooled | 0.983 | 0.000 | -5.48 | 3.113 |  |  |  |
|  |  | PEUE | EU | 0.996 | 0.000 | 0.73 | 0.000 | 0.389 | **0.001** | 0.93 |
|  |  |  | AMn | 0.992 | 0.000 | 0.77 | -0.001 |  |  |  |
|  |  |  | pooled | 0.989 | 0.000 | 0.71 | 0.002 |  |  |  |
| R_area_ | | RE | EU | 0.564 | 0.085 | -2.32 | 2.210 | **0.009** | - | - |
|  |  |  | AMn | 0.948 | 0.005 | -0.59 | 1.206 |  |  |  |
|  |  |  | pooled | 0.346 | 0.057 | -1.53 | 1.739 |  |  |  |
| CC_mass_ | | Payback time | EU | 0.100 | 0.541 | -23.59 | 11.664 | 0.814 | 0.174 | **0.006** |
|  |  |  | AMn | 0.060 | 0.692 | 19.75 | -4.669 |  |  |  |
|  |  |  | pooled | 0.213 | 0.154 | -13.24 | 7.684 |  |  |  |
| *g*_s_ | | *A*_mass_ | EU | 0.814 | 0.014 | 0.488 | 0.033 | 0.957 | 0.698 | **0.048** |
|  |  |  | AMn | 0.803 | 0.040 | 0.479 | 0.036 |  |  |  |
|  |  |  | pooled | 0.869 | 0.000 | 0.468 | 0.036 |  |  |  |
|  |  | IWUE | EU | 0.783 | 0.019 | -1.992 | 2.050 | 0.272 | 0.676 | **0.022** |
|  |  |  | AMn | 0.906 | 0.013 | -2.852 | 2.140 |  |  |  |
|  |  |  | pooled | 0.868 | 0.000 | -2.456 | 2.104 |  |  |  |
| *C_i_* | | *A*_mass_ | EU | 0.133 | 0.478 | 0.739 | -1.679 | 0.313 | 0.631 | **0.020** |
|  |  |  | AMn | 0.483 | 0.193 | 0.365 | -0.787 |  |  |  |
|  |  |  | pooled | 0.443 | 0.025 | 0.468 | -1.052 |  |  |  |
|  |  |  | EU | 0.098 | 0.545 | 5.032 | -10.748 | 0.288 | 0.363 | **0.039** |
|  |  | *A*_area_ | AMn | 0.346 | 0.296 | 2.265 | -4.119 |  |  |  |
|  |  |  | pooled | 0.295 | 0.084 | 2.913 | -5.667 |  |  |  |
| P_leaf_ | | P_leaf sheath_ | EU | 0.541 | 0.156 | -0.107 | 0.100 | **0.009** | - | - |
|  |  |  | AMn | 0.531 | 0.100 | 0.720 | 0.003 |  |  |  |
|  |  |  | pooled | 0.078 | 0.407 | 0.350 | 0.049 |  |  |  |
| P_stem_ | | P_leaf_ | EU | 0.994 | 0.000 | -0.809 | 0.261 | **0.008** | - | - |
|  |  |  | AMn | 0.911 | 0.003 | -1.430 | 0.333 |  |  |  |
|  |  |  | pooled | 0.912 | 0.000 | -1.015 | 0.283 |  |  |  |
|  |  | P_leaf sheath_ | EU | 0.466 | 0.204 | 7.568 | -0.499 | **0.038** | - | - |
|  |  |  | AMn | 0.809 | 0.015 | -1.986 | 0.340 |  |  |  |
|  |  |  | pooled | 0.303 | 0.080 | -2.902 | 0.426 |  |  |  |

Table S3 Results of standardized major axis (SMA) regression analysis between AM and EU. Significant results (P < 0.05) are shown in bold. Traits marked in red colour indicate relationships plotted in Fig.1. C_mass_ and C_area_: mass-and area based leaf carbon concentration; N_mass_ and N_area_: mass-and area based leaf nitrogen concentration; *A*_mass_ and *A*_area_: mass-and area based leaf light-saturated photosynthetic assimilation; R_area_: area based leaf respiration; *g*_s_: stomatal conductance; PNUE: leaf photosynthetic nitrogen use efficiency; IWUE: leaf intrinsic water use efficiency; CC_mass_: mass-based leaf construction cost; RE: leaf respiration efficiency; PEUE: leaf photosynthetic energy-use efficiency; P_leaf sheath_, P_leaf_, and P_stem_ are proportions of leaf sheath, leaf, and stem biomass to the total shoot biomass, respectively.

| Trait X | Trait Y | | group | r^2^ | *p* | Slope | Intercept | Slope homogeneity (S) | Shift in elevation (E) | Shift along the common slope (CS) |
| --- | --- | --- | --- | --- | --- | --- | --- | --- | --- | --- |
| C_mass_ | | PNUE | AM | 0.333 | 0.104 | -12.978 | 22.64 | 0.626 | **0.036** | 0.406 |
|  |  |  | EU | 0.217 | 0.352 | -9.788 | 17.27 |  |  |  |
|  |  |  | pooled | 0.070 | 0.259 | -11.36 | 19.90 |  |  |  |
|  |  | CC_mass_ | AM | 0.145 | 0.312 | 1.073 | -1.409 | 0.170 | **0.000** | 0.330 |
|  |  |  | EU | 0.805 | 0.015 | 0.589 | -0.599 |  |  |  |
|  |  |  | pooled | 0.341 | 0.007 | 1.106 | -1.463 |  |  |  |
| C_area_ | | PNUE | AM | 0.018 | 0.730 | -0.767 | 2.245 | 0.501 | **0.033** | 0.382 |
|  |  |  | EU | 0.035 | 0.723 | -1.184 | 2.846 |  |  |  |
|  |  |  | pooled | 0.002 | 0.871 | 1.219 | -0.958 |  |  |  |
|  |  | CC_mass_ | AM | 0.008 | 0.816 | -0.063 | 0.480 | 0.845 | **0.009** | 0.373 |
|  |  |  | EU | 0.322 | 0.241 | 0.071 | 0.269 |  |  |  |
|  |  |  | pooled | 0.001 | 0.923 | 0.119 | 0.189 |  |  |  |
|  |  | CC_area_ | AM | 0.989 | 0.000 | 0.9927 | 0.489 | 0.190 | **0.001** | 0.524 |
|  |  |  | EU | 0.996 | 0.000 | 1.063 | 0.384 |  |  |  |
|  |  |  | pooled | 0.975 | 0.000 | 1.017 | 0.451 |  |  |  |
| N_mass_ | | *A*_area_ | AM | 0.223 | 0.199 | -2.107 | 2.468 | 0.606 | **0.016** | 0.836 |
|  |  |  | EU | 0.613 | 0.066 | 2.730 | -0.249 |  |  |  |
|  |  |  | pooled | 0.351 | 0.006 | 2.074 | 0.163 |  |  |  |
|  |  | RE | AM | 0.055 | 0.542 | -4.341 | 3.387 | 0.915 | **0.027** | 0.835 |
|  |  |  | EU | 0.452 | 0.144 | 4.042 | -1.310 |  |  |  |
|  |  |  | pooled | 0.085 | 0.213 | 2.863 | -0.571 |  |  |  |
|  |  | Payback time | AM | 0.004 | 0.872 | -1.991 | 3.693 | 0.870 | **0.039** | 0.893 |
|  |  |  | EU | 0.395 | 0.182 | -2.178 | 3.862 |  |  |  |
|  |  |  | pooled | 0.432 | 0.002 | -1.845 | 3.640 |  |  |  |
|  |  | PEUE | AM | 0.004 | 0.878 | 0.291 | -0.091 | 0.989 | **0.049** | 0.938 |
|  |  |  | EU | 0.389 | 0.186 | 0.294 | -0.101 |  |  |  |
|  |  |  | pooled | 0.404 | 0.003 | 0.241 | -0.067 |  |  |  |
| N_area_ | | CC_mass_ | AM | 0.046 | 0.580 | 0.112 | 0.323 | 0.260 | **0.035** | 0.233 |
|  |  |  | EU | 0.481 | 0.126 | 0.059 | 0.353 |  |  |  |
|  |  |  | pooled | 0.280 | 0.016 | 0.105 | 0.327 |  |  |  |
|  |  | CC_area_ | AM | 0.699 | 0.005 | 1.748 | 1.209 | **0.041** | - | - |
|  |  |  | EU | 0.786 | 0.018 | 0.880 | 1.629 |  |  |  |
|  |  |  | pooled | 0.417 | 0.002 | 0.895 | 1.636 |  |  |  |
| *A*_area_ | | CC_mass_ | AM | 0.425 | 0.057 | -0.072 | 0.475 | 0.165 | 0.812 | **0.010** |
|  |  |  | EU | 0.279 | 0.281 | 0.034 | 0.339 |  |  |  |
|  | |  | pooled | 0.084 | 0.215 | 0.057 | 0.305 |  |  |  |
| *A*_mass_ | | CC_mass_ | AM | 0.388 | 0.073 | -0.438 | 0.418 | 0.275 | 0.561 | **0.033** |
|  |  |  | EU | 0.128 | 0.486 | 0.230 | 0.362 |  |  |  |
|  |  |  | pooled | 0.121 | 0.132 | 0.361 | 0.348 |  |  |  |
|  |  | Payback time | AM | 0.989 | 0.000 | -5.707 | 3.128 | 0.347 | **0.000** | 0.334 |
|  |  |  | EU | 0.997 | 0.000 | -5.435 | 3.113 |  |  |  |
|  |  |  | pooled | 0.987 | 0.000 | -5.620 | 3.122 |  |  |  |
|  |  | PEUE | AM | 0.989 | 0.000 | 0.835 | -0.009 | **0.035** |  |  |
|  |  |  | EU | 0.996 | 0.000 | 0.733 | 0 |  |  |  |
|  |  |  | pooled | 0.989 | 0.000 | 0.734 | 0 |  |  |  |
| CC_mass_ | | Payback time | AM | 0.487 | 0.037 | 13.04 | -2.328 | 0.291 | 0.703 | **0.025** |
|  |  |  | EU | 0.100 | 0.541 | -23.59 | 11.664 |  |  |  |
|  |  |  | pooled | 0.068 | 0.268 | -15.57 | 8.537 |  |  |  |
|  |  | PEUE | AM | 0.490 | 0.036 | -1.908 | 0.790 | 0.373 | 0.624 | **0.030** |
|  |  |  | EU | 0.087 | 0.570 | 3.181 | -1.153 |  |  |  |
|  |  |  | pooled | 0.061 | 0.293 | 2.034 | -0.707 |  |  |  |
| P _stem biomass_ | | P _leaf biomass_ | AM | 0.661 | 0.008 | -1.014 | 0.272 | 0.187 | **0.000** | 0.558 |
|  |  |  | EU | 0.911 | 0.003 | -0.699 | 0.233 |  |  |  |
|  |  |  | pooled | 0.667 | 0.000 | -0.835 | 0.250 |  |  |  |
|  |  | P _leaf sheath biomass_ | AM | 0.169 | 0.272 | -0.664 | 0.203 | 0.525 | **0.011** | **0.006** |
|  |  |  | EU | 0.809 | 0.015 | -0.504 | 0.171 |  |  |  |
|  |  |  | pooled | 0.447 | 0.001 | -0.653 | 0.200 |  |  |  |
| P _leaf biomass_ | | P _leaf sheath biomass_ | AM | 0.037 | 0.618 | -0.655 | 0.180 | 0.863 | 0.001 | 0.044 |
|  |  |  | EU | 0.531 | 0.100 | 0.720 | 0.003 |  |  |  |
|  |  |  | pooled | 0.014 | 0.620 | 0.782 | 0.004 |  |  |  |
| *g*_s_ | | IWUE | AM | 0.480 | 0.039 | -2.875 | 1.992 | 0.324 | 0.900 | 0.119 |
|  |  |  | EU | 0.783 | 0.019 | -1.992 | 1.909 |  |  |  |
|  |  |  | pooled | 0.866 | 0.000 | -2.494 | 2.109 |  |  |  |
|  |  | *A*_mass_ | AM | 0.081 | 0.458 | 0.437 | 0.036 | 0.842 | 0.313 | 0.230 |
|  |  |  | EU | 0.814 | 0.014 | 0.488 | 0.033 |  |  |  |
|  |  |  | pooled | 0.722 | 0.000 | 0.418 | 0.039 |  |  |  |

**Table S4** Results of standardized major axis (SMA) regression analysis between AM and AMn. Significant results (P < 0.05) are shown in bold. Traits marked in red colour indicate relationships plotted in Fig.1. C_mass_ and C_area_: mass-and area based leaf carbon concentration; N_mass_ and N_area_: mass-and area- based leaf nitrogen concentration; *A*_mass_ and *A*_area_: mass-and area- based leaf light-saturated photosynthetic assimilation; R_area_: area based leaf respiration; *g*_s_: stomatal conductance; *C_i_*: intercellular CO_2_ concentration; PNUE: leaf photosynthetic nitrogen use efficiency; IWUE: leaf intrinsic water use efficiency; CC_mass_ and CC_area_: mass- and area-based leaf construction cost; RE: leaf respiration efficiency; PEUE: leaf photosynthetic energy-use efficiency; P_leaf sheath_, P_leaf_, and P_stem_ are proportions of leaf sheath, leaf, and stem biomass to the total shoot biomass, respectively.

| Trait X | Trait Y | | group | r^2^ | *p* | Slope | Intercept | Slope homogeneity (S) | Shift in elevation (E) | Shift along the common slope (CS) |
| --- | --- | --- | --- | --- | --- | --- | --- | --- | --- | --- |
| Chl *a*_area_ | | *A*_area_ | AM | 0.341 | 0.099 | -1.069 | 1.998 | 0.498 | **0.003** | 0.640 |
|  |  |  | AMn | 0.354 | 0.290 | 0.713 | 0.805 |  |  |  |
|  |  |  | pooled | 0.263 | 0.061 | 1.216 | 0.542 |  |  |  |
|  |  | *A*_mass_ | AM | 0.008 | 0.821 | 0.177 | -0.021 | 0.430 | **0.032** | **0.004** |
|  |  |  | AMn | 0.575 | 0.137 | 0.115 | 0.007 |  |  |  |
|  |  |  | pooled | 0.461 | 0.008 | 0.201 | -0.038 |  |  |  |
| Chl *a*_mass_ | | *A*_area_ | AM | 0.002 | 0.913 | 4.262 | 0.865 | 0.516 | 0.102 | **0.004** |
|  |  |  | AMn | 0.456 | 0.211 | 2.805 | 0.957 |  |  |  |
|  |  |  | pooled | 0.450 | 0.009 | 4.679 | 0.808 |  |  |  |
|  |  | *A*_mass_ | AM | 0.190 | 0.241 | -0.706 | -0.338 | 0.351 | 0.053 | **0.003** |
|  |  |  | AMn | 0.652 | 0.099 | 0.452 | 0.031 |  |  |  |
|  |  |  | pooled | 0.389 | 0.017 | 0.774 | 0.006 |  |  |  |
| Chl *b*_area_ | | *A*_area_ | AM | 0.224 | 0.198 | -1.594 | 1.834 | 0.512 | **0.001** | 0.319 |
|  |  |  | AMn | 0.346 | 0.297 | 1.081 | 0.923 |  |  |  |
|  |  |  | pooled | 0.369 | 0.021 | 1.663 | 0.782 |  |  |  |
|  |  | *A*_mass_ | AM | 0.204 | 0.222 | 0.264 | 0.006 | 0.462 | 0.137 | **0.002** |
|  |  |  | AMn | 0.572 | 0.139 | 0.174 | 0.026 |  |  |  |
|  |  |  | pooled | 0.644 | 0.001 | 0.275 | 0.001 |  |  |  |
| Chl *b*_mass_ | | *A*_area_ | AM | 0.131 | 0.338 | 12.504 | 0.833 | 0.397 | 0.409 | **0.002** |
|  |  |  | AMn | 0.549 | 0.152 | 7.603 | 0.980 |  |  |  |
|  |  |  | pooled | 0.669 | 0.000 | 11.17 | 0.880 |  |  |  |
|  |  | *A*_mass_ | AM | 0.024 | 0.688 | 2.071 | 0.010 | 0.329 | 0.179 | **0.001** |
|  |  |  | AMn | 0.741 | 0.061 | 1.226 | 0.035 |  |  |  |
|  |  |  | pooled | 0.669 | 0.000 | 1.848 | 0.017 |  |  |  |
| Total Chl_area_ | | *A*_area_ | AM | 0.355 | 0.090 | -1.083 | 2.113 | 0.444 | **0.003** | 0.228 |
|  |  |  | AMn | 0.358 | 0.287 | 0.667 | 0.772 |  |  |  |
|  |  |  | pooled | 0.306 | 0.040 | 1.124 | 0.492 |  |  |  |
|  |  | *A*_mass_ | AM | 0.036 | 0.625 | 0.179 | -0.040 | 0.388 | 0.061 | **0.004** |
|  |  |  | AMn | 0.581 | 0.134 | 0.108 | 0.001 |  |  |  |
|  |  |  | pooled | 0.528 | 0.003 | 0.186 | -0.047 |  |  |  |
| Total Chl_mass_ | | *A*_area_ | AM | 0.018 | 0.734 | 4.340 | 0.722 | 0.287 | 0.263 | **0.004** |
|  |  |  | AMn | 0.484 | 0.192 | 2.268 | 0.950 |  |  |  |
|  |  |  | pooled | 0.543 | 0.003 | 3.771 | 0.792 |  |  |  |
|  |  | *A*_mass_ | AM | 0.137 | 0.326 | -0.719 | 0.190 | 0.201 | 0.136 | **0.003** |
|  |  |  | AMn | 0.679 | 0.086 | 0.366 | 0.030 |  |  |  |
|  |  |  | pooled | 0.493 | 0.005 | 0.624 | 0.003 |  |  |  |
| C_mass_ | | *A*_area_ | AM | 0.085 | 0.445 | -14.81 | 25.99 | 0.842 | **0.000** | 0.130 |
|  |  |  | AMn | 0.001 | 0.963 | 12.83 | -20.13 |  |  |  |
|  |  |  | pooled | 0.108 | 0.251 | 20.94 | -33.58 |  |  |  |
|  |  | *A*_mass_ | AM | 0.743 | 0.003 | -2.452 | 4.176 | 0.801 | **0.000** | 0.109 |
|  |  |  | AMn | 0.058 | 0.696 | 2.069 | -3.368 |  |  |  |
|  |  |  | pooled | 0.069 | 0.364 | 3.464 | -5.683 |  |  |  |
|  |  | RE | AM | 0.349 | 0.094 | -30.51 | 51.86 | 0.184 | **0.003** | 0.421 |
|  |  |  | AMn | 0.094 | 0.617 | 11.29 | -17.88 |  |  |  |
|  |  |  | pooled | 0.034 | 0.530 | 26.71 | -43.48 |  |  |  |
|  |  | PNUE | AM | 0.333 | 0.104 | -12.978 | 22.64 | **0.042** | - | - |
|  |  |  | AMn | 0.946 | 0.005 | -6.023 | 10.98 |  |  |  |
|  |  |  | pooled | 0.013 | 0.697 | -11.53 | 20.19 |  |  |  |
|  |  | IWUE | AM | 0.164 | 0.279 | -16.12 | 28.65 | 0.718 | 0.058 | **0.000** |
|  |  |  | AMn | 0.000 | 0.993 | 12.31 | -18.55 |  |  |  |
|  |  |  | pooled | 0.237 | 0.077 | -23.32 | 40.66 |  |  |  |
|  |  | CC_mass_ | AM | 0.145 | 0.312 | 1.073 | -1.409 | 0.460 | 0.194 | **0.023** |
|  |  |  | AMn | 0.501 | 0.181 | 0.700 | -0.791 |  |  |  |
|  |  |  | pooled | 0.440 | 0.010 | 1.077 | -1.416 |  |  |  |
|  |  | CC_area_ | AM | 0.170 | 0.270 | 16.809 | -25.924 | **0.040** | - | - |
|  |  |  | AMn | 0.364 | 0.281 | -3.667 | 8.166 |  |  |  |
|  |  |  | pooled | 0.050 | 0.444 | 10.01 | -14.59 |  |  |  |
|  |  | Payback time | AM | 0.700 | 0.005 | 14.00 | -20.71 | 0.988 | **0.001** | 0.180 |
|  |  |  | AMn | 0.027 | 0.791 | -13.83 | 25.71 |  |  |  |
|  |  |  | pooled | 0.057 | 0.411 | -19.84 | 35.68 |  |  |  |
|  |  | PEUE | AM | 0.700 | 0.005 | -2.047 | 3.478 | 0.739 | **0.001** | 0.234 |
|  |  |  | AMn | 0.028 | 0.786 | 1.603 | -2.611 |  |  |  |
|  |  |  | pooled | 0.043 | 0.475 | 2.553 | -4.188 |  |  |  |
| C_area_ | | *A*_area_ | AM | 0.429 | 0.055 | 0.875 | -0.080 | **0.026** | - | - |
|  |  |  | AMn | 0.195 | 0.457 | 3.924 | -5.044 |  |  |  |
|  |  |  | pooled | 0.174 | 0.137 | 2.122 | -2.113 |  |  |  |
|  |  | *A*_mass_ | AM | 0.268 | 0.153 | -0.145 | 0.323 | 0.063 | **0.000** | 0.007 |
|  |  |  | AMn | 0.026 | 0.796 | 0.633 | -0.937 |  |  |  |
|  |  |  | pooled | 0.003 | 0.850 | -0.351 | 0.646 |  |  |  |
|  |  | *R*_area_ | AM | 0.166 | 0.276 | 1.106 | -1.260 | **0.024** | - | - |
|  |  |  | AMn | 0.304 | 0.335 | 5.904 | -8.897 |  |  |  |
|  |  |  | pooled | 0.116 | 0.234 | 1.704 | -2.214 |  |  |  |
|  |  | *R*_mass_ | AM | 0.062 | 0.520 | -0.040 | 0.075 | **0.026** | - | - |
|  |  |  | AMn | 0.202 | 0.448 | 0.212 | -0.327 |  |  |  |
|  |  |  | pooled | 0.007 | 0.778 | -0.062 | 0.110 |  |  |  |
|  |  | RE | AM | 0.008 | 0.819 | -1.802 | 3.918 | 0.356 | **0.001** | 0.068 |
|  |  |  | AMn | 0.247 | 0.395 | -3.454 | 6.411 |  |  |  |
|  |  |  | pooled | 0.001 | 0.938 | 2.706 | -3.349 |  |  |  |
|  |  | PNUE | AM | 0.018 | 0.730 | -0.767 | 2.245 | 0.192 | 0.070 | **0.013** |
|  |  |  | AMn | 0.380 | 0.268 | 1.842 | -1.973 |  |  |  |
|  |  |  | pooled | 0.006 | 0.800 | 1.168 | -0.873 |  |  |  |
|  |  | IWUE | AM | 0.041 | 0.603 | -0.952 | 3.321 | 0.088 | **0.001** | **0.000** |
|  |  |  | AMn | 0.010 | 0.876 | -3.764 | 7.924 |  |  |  |
|  |  |  | pooled | 0.046 | 0.462 | -2.363 | 5.623 |  |  |  |
|  |  | CC_mass_ | AM | 0.008 | 0.816 | -0.063 | 0.480 | 0.106 | **0.005** | 0.099 |
|  |  |  | AMn | 0.078 | 0.649 | -0.214 | 0.715 |  |  |  |
|  |  |  | pooled | 0.001 | 0.914 | 0.109 | 0.202 |  |  |  |
|  |  | CC_area_ | AM | 0.989 | 0.000 | 0.9927 | 0.489 | 0.440 | **0.036** | 0.427 |
|  |  |  | AMn | 0.948 | 0.005 | 0.122 | 0.279 |  |  |  |
|  |  |  | pooled | 0.981 | 0.000 | 1.015 | 0.453 |  |  |  |
|  |  | Payback time | AM | 0.215 | 0.209 | 0.827 | 1.284 | **0.039** | - | - |
|  |  |  | AMn | 0.037 | 0.756 | -4.230 | 9.455 |  |  |  |
|  |  |  | pooled | 0.001 | 0.908 | 2.010 | -0.572 |  |  |  |
|  |  | PEUE | AM | 0.213 | 0.212 | -0.121 | 0.261 | 0.076 | **0.001** | **0.020** |
|  |  |  | AMn | 0.035 | 0.765 | 0.490 | -0.727 |  |  |  |
|  |  |  | pooled | 0.004 | 0.838 | -0.259 | 0.477 |  |  |  |
| N_mass_ | | *A*_area_ | AM | 0.223 | 0.199 | -2.107 | 2.469 | 0.267 | 0.096 | **0.006** |
|  |  |  | AMn | 0.598 | 0.125 | 1.135 | 0.637 |  |  |  |
|  |  |  | pooled | 0.383 | 0.018 | 2.020 | 0.212 |  |  |  |
|  |  | *A*_mass_ | AM | 0.020 | 0.718 | 0.349 | -0.099 | 0.205 | **0.004** | **0.005** |
|  |  |  | AMn | 0.799 | 0.041 | 0.183 | -0.020 |  |  |  |
|  |  |  | pooled | 0.555 | 0.002 | 0.334 | -0.093 |  |  |  |
|  |  | RE | AM | 0.055 | 0.542 | -4.341 | 3.387 | 0.058 | **0.001** | 0.793 |
|  |  |  | AMn | 0.150 | 0.519 | -0.999 | 1.410 |  |  |  |
|  |  |  | pooled | 0.064 | 0.384 | 2.577 | -0.384 |  |  |  |
|  |  | PNUE | AM | 0.254 | 0.166 | -1.846 | 2.019 | 0.061 | **0.000** | 0.893 |
|  |  |  | AMn | 0.398 | 0.254 | -0.533 | 1.234 |  |  |  |
|  |  |  | pooled | 0.009 | 0.753 | 1.112 | 0.407 |  |  |  |
|  |  | IWUE | AM | 0.013 | 0.768 | 2.293 | 0.548 | 0.269 | 0.094 | **0.002** |
|  |  |  | AMn | 0.330 | 0.311 | -1.088 | 2.474 |  |  |  |
|  |  |  | pooled | 0.365 | 0.022 | -2.250 | 3.034 |  |  |  |
|  |  | CC_mass_ | AM | 0.268 | 0.153 | 0.153 | 0.296 | 0.223 | 0.881 | **0.012** |
|  |  |  | AMn | 0.021 | 0.814 | 0.062 | 0.342 |  |  |  |
|  |  |  | pooled | 0.297 | 0.044 | 0.104 | 0.322 |  |  |  |
|  |  | CC_area_ | AM | 0.263 | 0.158 | -2.391 | 3.382 | **0.019** | - | - |
|  |  |  | AMn | 0.065 | 0.679 | -0.324 | 2.232 |  |  |  |
|  |  |  | pooled | 0.011 | 0.721 | -0.966 | 2.589 |  |  |  |
|  |  | Payback time | AM | 0.004 | 0.872 | -1.991 | 3.693 | 0.318 | 0.051 | **0.013** |
|  |  |  | AMn | 0.746 | 0.059 | -1.223 | 3.331 |  |  |  |
|  |  |  | pooled | 0.558 | 0.002 | -1.914 | 3.662 |  |  |  |
|  |  | PEUE | AM | 0.004 | 0.878 | 0.291 | -0.091 | 0.175 | **0.049** | **0.010** |
|  |  |  | AMn | 0.746 | 0.059 | 0.142 | -0.018 |  |  |  |
|  |  |  | pooled | 0.514 | 0.004 | 0.246 | -0.068 |  |  |  |
| N_area_ | | *A*_area_ | AM | 0.249 | 0.171 | 1.540 | 0.554 | 0.645 | **0.005** | **0.004** |
|  |  |  | AMn | 0.802 | 0.040 | 1.262 | 0.632 |  |  |  |
|  |  |  | pooled | 0.659 | 0.000 | 1.951 | 0.337 |  |  |  |
|  |  | *A*_mass_ | AM | 0.228 | 0.194 | -0.255 | 0.218 | 0.569 | **0.007** | **0.002** |
|  |  |  | AMn | 0.905 | 0.013 | 0.204 | -0.021 |  |  |  |
|  |  |  | pooled | 0.395 | 0.016 | 0.323 | -0.072 |  |  |  |
|  |  | *R*_mass_ | AM | 0.002 | 0.903 | 0.070 | -0.023 | 0.964 | **0.047** | 0.392 |
|  |  |  | AMn | 0.471 | 0.200 | 0.068 | -0.019 |  |  |  |
|  |  |  | pooled | 0.096 | 0.280 | 0.057 | -0.016 |  |  |  |
|  |  | RE | AM | 0.051 | 0.557 | -3.174 | 2.612 | 0.145 | **0.000** | 0.598 |
|  |  |  | AMn | 0.282 | 0.357 | -1.111 | 1.414 |  |  |  |
|  |  |  | pooled | 0.057 | 0.411 | 2.488 | -0.224 |  |  |  |
|  |  | PNUE | AM | 0.230 | 0.191 | -1.350 | 1.690 | 0.234 | **0.000** | 0.773 |
|  |  |  | AMn | 0.207 | 0.442 | -0.593 | 1.236 |  |  |  |
|  |  |  | pooled | 0.020 | 0.631 | 1.074 | 0.476 |  |  |  |
|  |  | IWUE | AM | 0.018 | 0.734 | -1.676 | 2.632 | 0.605 | **0.035** | **0.001** |
|  |  |  | AMn | 0.392 | 0.259 | -1.211 | 2.479 |  |  |  |
|  |  |  | pooled | 0.436 | 0.010 | -2.172 | 2.894 |  |  |  |
|  |  | CC_mass_ | AM | 0.046 | 0.580 | 0.112 | 0.323 | 0.513 | 0.701 | **0.004** |
|  |  |  | AMn | 0.001 | 0.996 | 0.069 | 0.342 |  |  |  |
|  |  |  | pooled | 0.223 | 0.089 | 0.100 | 0.328 |  |  |  |
|  |  | CC_area_ | AM | 0.669 | 0.005 | 1.748 | 1.209 | **0.042** | - | - |
|  |  |  | AMn | 0.001 | 0.958 | -0.361 | 2.233 |  |  |  |
|  |  |  | pooled | 0.272 | 0.056 | 0.933 | 1.624 |  |  |  |
|  |  | Payback time | AM | 0.217 | 0.206 | 1.456 | 1.883 | 0.877 | 0.056 | **0.007** |
|  |  |  | AMn | 0.870 | 0.021 | -1.360 | 3.336 |  |  |  |
|  |  |  | pooled | 0.418 | 0.013 | -1.848 | 3.543 |  |  |  |
|  |  | PEUE | AM | 0.216 | 0.207 | -0.213 | 0.174 | 0.480 | **0.048** | **0.005** |
|  |  |  | AMn | 0.867 | 0.021 | 0.158 | -0.018 |  |  |  |
|  |  |  | pooled | 0.365 | 0.022 | 0.238 | -0.053 |  |  |  |
| *g*_s_ | | *A*_area_ | AM | 0.636 | 0.010 | 2.642 | 0.989 | 0.743 | 0.281 | **0.000** |
|  |  |  | AMn | 0.810 | 0.037 | 2.973 | 0.985 |  |  |  |
|  |  |  | pooled | 0.906 | 0.000 | 2.369 | 1.026 |  |  |  |
|  |  | *A*_mass_ | AM | 0.081 | 0.458 | 0.437 | 0.036 | 0.836 | 0.396 | **0.000** |
|  |  |  | AMn | 0.803 | 0.040 | 0.479 | 0.036 |  |  |  |
|  |  |  | pooled | 0.775 | 0.000 | 0.392 | 0.042 |  |  |  |
|  |  | IWUE | AM | 0.480 | 0.039 | -2.875 | 2.158 | 0.991 | 0.554 | **0.000** |
|  |  |  | AMn | 0.906 | 0.013 | -2.852 | 2.140 |  |  |  |
|  |  |  | pooled | 0.904 | 0.000 | -2.638 | 2.126 |  |  |  |
| *C_i_* | | *A*_area_ | AM | 0.019 | 0.721 | -2.407 | 7.101 | 0.924 | 0.978 | **0.000** |
|  |  |  | AMn | 0.346 | 0.296 | 2.265 | -4.119 |  |  |  |
|  |  |  | pooled | 0.563 | 0.002 | 2.355 | -4.330 |  |  |  |
|  |  | *A*_mass_ | AM | 0.046 | 0.580 | -0.399 | 1.048 | 0.882 | 0.929 | **0.000** |
|  |  |  | AMn | 0.483 | 0.193 | 0.365 | -0.787 |  |  |  |
|  |  |  | pooled | 0.567 | 0.002 | 0.390 | -0.844 |  |  |  |
|  |  | *A*_area_ | AM | 0.596 | 0.015 | 1.273 | 0.342 | 0.413 | 0.348 | **0.000** |
|  |  |  | AMn | 0.869 | 0.021 | 0.967 | 0.587 |  |  |  |
|  |  |  | pooled | 0.913 | 0.000 | 0.938 | 0.602 |  |  |  |
|  |  | *A*_mass_ | AM | 0.000 | 0.992 | -0.211 | 0.253 | 0.531 | 0.632 | **0.000** |
|  |  |  | AMn | 0.825 | 0.033 | 0.156 | -0.028 |  |  |  |
|  |  |  | pooled | 0.743 | 0.000 | 0.155 | -0.028 |  |  |  |
| *A*_area_ | | RE | AM | 0.147 | 0.308 | 2.061 | -1.699 | 0.119 | **0.000** | 0.714 |
|  |  |  | AMn | 0.628 | 0.110 | -0.880 | 1.971 |  |  |  |
|  |  |  | pooled | 0.342 | 0.028 | 1.275 | -0.654 |  |  |  |
|  |  | PNUE | AM | 0.270 | 0.152 | 0.876 | -0.144 | 0.394 | 0.362 | **0.000** |
|  |  |  | AMn | 0.000 | 0.984 | -0.469 | 1.533 |  |  |  |
|  |  |  | pooled | 0.479 | 0.006 | 0.551 | 0.290 |  |  |  |
|  |  | IWUE | AM | 0.026 | 0.677 | -1.088 | 3.234 | 0.823 | 0.692 | **0.000** |
|  |  |  | AMn | 0.553 | 0.150 | -0.959 | 3.085 |  |  |  |
|  |  |  | pooled | 0.690 | 0.000 | -1.113 | 3.269 |  |  |  |
|  |  | CC_mass_ | AM | 0.425 | 0.057 | -0.072 | 0.475 | 0.686 | **0.000** | 0.463 |
|  |  |  | AMn | 0.068 | 0.673 | -0.055 | 0.440 |  |  |  |
|  |  |  | pooled | 0.145 | 0.179 | 0.051 | 0.311 |  |  |  |
|  |  | CC_area_ | AM | 0.393 | 0.071 | 1.135 | 0.580 | **0.049** | - | - |
|  |  |  | AMn | 0.093 | 0.617 | 0.286 | 1.721 |  |  |  |
|  |  |  | pooled | 0.205 | 0.104 | 0.478 | 1.463 |  |  |  |
|  |  | Payback time | AM | 0.130 | 0.340 | -0.945 | 3.861 | 0.728 | 0.465 | **0.000** |
|  |  |  | AMn | 0.930 | 0.008 | -1.078 | 4.018 |  |  |  |
|  |  |  | pooled | 0.793 | 0.000 | -0.947 | 3.862 |  |  |  |
|  |  | PEUE | AM | 0.131 | 0.338 | 0.138 | -0.116 | 0.784 | 0.708 | **0.000** |
|  |  |  | AMn | 0.927 | 0.009 | 0.125 | -0.097 |  |  |  |
|  |  |  | pooled | 0.770 | 0.000 | 0.122 | -0.094 |  |  |  |
| *A*_mass_ | | RE | AM | 0.322 | 0.111 | 12.442 | -0.103 | 0.163 | 0.540 | **0.000** |
|  |  |  | AMn | 0.534 | 0.161 | -5.458 | 1.299 |  |  |  |
|  |  |  | pooled | 0.416 | 0.013 | 7.710 | 0.333 |  |  |  |
|  |  | PNUE | AM | 0.609 | 0.013 | 5.292 | 0.535 | 0.386 | 0.260 | **0.000** |
|  |  |  | AMn | 0.048 | 0.724 | -2.911 | 1.175 |  |  |  |
|  |  |  | pooled | 0.535 | 0.003 | 3.328 | 0.717 |  |  |  |
|  |  | IWUE | AM | 0.011 | 0.792 | 6.572 | 1.198 | 0.850 | 0.711 | **0.000** |
|  |  |  | AMn | 0.656 | 0.096 | -5.949 | 2.353 |  |  |  |
|  |  |  | pooled | 0.642 | 0.001 | -6.731 | 2.407 |  |  |  |
|  |  | CC_mass_ | AM | 0.388 | 0.073 | -0.438 | 0.418 | 0.690 | **0.000** | 0.441 |
|  |  |  | AMn | 0.020 | 0.820 | -0.339 | 0.398 |  |  |  |
|  |  |  | pooled | 0.171 | 0.142 | 0.311 | 0.351 |  |  |  |
|  |  | CC_area_ | AM | 0.296 | 0.130 | -6.855 | 2.704 | 0.077 | **0.001** | **0.028** |
|  |  |  | AMn | 0.001 | 0.961 | 1.772 | 1.939 |  |  |  |
|  |  |  | pooled | 0.000 | 0.966 | -2.890 | 2.320 |  |  |  |
|  |  | Payback time | AM | 0.989 | 0.000 | -5.707 | 3.128 | 0.076 | 0.197 | **0.000** |
|  |  |  | AMn | 0.989 | 0.001 | -6.685 | 3.195 |  |  |  |
|  |  |  | pooled | 0.990 | 0.000 | -5.727 | 3.129 |  |  |  |
|  |  | PEUE | AM | 0.982 | 0.000 | 0.835 | -0.009 | 0.307 | **0.001** | **0.000** |
|  |  |  | AMn | 0.992 | 0.000 | 0.775 | -0.002 |  |  |  |
|  |  |  | pooled | 0.992 | 0.000 | 0.737 | 0.000 |  |  |  |
| *R*_area_ | | RE | AM | 0.659 | 0.008 | -1.630 | 1.865 | **0.003** | - | - |
|  |  |  | AMn | 0.948 | 0.005 | -0.585 | 1.206 |  |  |  |
|  |  |  | pooled | 0.306 | 0.040 | -1.588 | 1.800 |  |  |  |
| *R*_mass_ | | RE | AM | 0-715 | 0.004 | -45.28 | 1.539 | **0.001** | - | -- |
|  |  |  | AMn | 0.960 | 0.003 | -16.27 | 1.093 |  |  |  |
|  |  |  | pooled | 0.381 | 0.019 | -43.99 | 1.486 |  |  |  |
| CC_mass_ | | Payback time | AM | 0.487 | 0.037 | 13.04 | -2.328 | 0.558 | **0.000** | 0.515 |
|  |  |  | AMn | 0.060 | 0.692 | 19.75 | -4.669 |  |  |  |
|  |  |  | pooled | 0.120 | 0.226 | -18.43 | 9.591 |  |  |  |
|  |  | PEUE | AM | 0.490 | 0.036 | -1.908 | 0.790 | 0.782 | **0.000** | 0.601 |
|  |  |  | AMn | 0.054 | 0.707 | -2.288 | 0.909 |  |  |  |
|  |  |  | pooled | 0.109 | 0.249 | 2.371 | -0.831 |  |  |  |
| CC_area_ | | Payback time | AM | 0.248 | 0.173 | 0.833 | 0.877 | 0.061 | **0.000** | **0.030** |
|  |  |  | AMn | 0.002 | 0.937 | -3.772 | 10.507 |  |  |  |
|  |  |  | pooled | 0.000 | 1.000 | 1.982 | -1.469 |  |  |  |
|  |  | PEUE | AM | 0.245 | 0.175 | -0.122 | 0.321 | 0.102 | **0.000** | **0.034** |
|  |  |  | AMn | 0.002 | 0.941 | 0.437 | -0.849 |  |  |  |
|  |  |  | pooled | 0.001 | 0.930 | -0.255 | 0.592 |  |  |  |
| P _leaf_ | | P _leaf sheath_ | AM | 0.037 | 0.618 | -0.655 | 0.181 | **0.012** | - | - |
|  |  |  | AMn | 0.541 | 0.156 | -0.107 | 0.100 |  |  |  |
|  |  |  | pooled | 0.023 | 0.606 | 0.730 | 0.014 |  |  |  |
| P _stem_ | | P _leaf_ | AM | 0.661 | 0.008 | -0.987 | 0.268 | 0.401 | **0.000** | **0.044** |
|  |  |  | AMn | 0.994 | 0.000 | -0.809 | 0.261 |  |  |  |
|  |  |  | pooled | 0.707 | 0.000 | -1.182 | 0.295 |  |  |  |
|  |  | P _leaf sheath_ | AM | 0.169 | 0.272 | -1.507 | 0.306 | **0.022** | - | - |
|  |  |  | AMn | 0.466 | 0.204 | 7.568 | -0.499 |  |  |  |
|  |  |  | pooled | 0.437 | 0.010 | -1.621 | 0.317 |  |  |  |

**Table S5** Factor loadings in PCA. *A*: leaf light-saturated photosynthetic assimilation; *g*_s_: stomatal conductance; *E*: transpiration rate; *C_i_*: intercellular CO_2_ concentration; PNUE: leaf photosynthetic nitrogen use efficiency; IWUE: leaf intrinsic water use efficiency; CC_mass_: mass-based leaf construction cost; PEUE: leaf photosynthetic energy-use efficiency; LDMC: leaf dry matter content; P_stem_ and P_leaf sheath_ are proportions of stem and leaf biomass to the total shoot biomass, respectively.

| **Attribute** | **Axis_1** | | **Axis_2** | | **Axis_3** | | **Axis_4** | |
| --- | --- | --- | --- | --- | --- | --- | --- | --- |
| **-** | Corr. | % (Tot. %) | Corr. | % (Tot. %) | Corr. | % (Tot. %) | Corr. | % (Tot. %) |
| *A*_mass_ | 0.930 | *86 % (86 %)* | -0.188 | *4 % (90 %)* | 0.076 | *1 % (91 %)* | 0.226 | *5 % (96 %)* |
| *gs* | 0.921 | *85 % (85 %)* | -0.299 | *9 % (94 %)* | -0.091 | *1 % (95 %)* | 0.052 | *0 % (95 %)* |
| *E* | 0.918 | *84 % (84 %)* | -0.248 | *6 % (90 %)* | -0.069 | *0 % (91 %)* | 0.073 | *1 % (91 %)* |
| Payback time | -0.917 | *84 % (84 %)* | 0.253 | *6 % (91 %)* | -0.088 | *1 % (91 %)* | -0.234 | *5 % (97 %)* |
| PEUE | 0.904 | *82 % (82 %)* | -0.271 | *7 % (89 %)* | 0.079 | *1 % (90 %)* | 0.237 | *6 % (95 %)* |
| IWUE | -0.872 | *76 % (76 %)* | 0.192 | *4 % (80 %)* | 0.245 | *6 % (86 %)* | 0.063 | *0 % (86 %)* |
| *A*_area_ | 0.867 | *75 % (75 %)* | -0.283 | *8 % (83 %)* | 0.147 | *2 % (85 %)* | 0.156 | *2 % (88 %)* |
| *C_i_* | 0.814 | *66 % (66 %)* | -0.081 | *1 % (67 %)* | -0.323 | *10 % (77 %)* | -0.154 | *2 % (80 %)* |
| C:N | -0.768 | *59 % (59 %)* | -0.474 | *22 % (81 %)* | -0.305 | *9 % (91 %)* | -0.206 | *4 % (95 %)* |
| N_mass_ | 0.763 | *58 % (58 %)* | 0.496 | *25 % (83 %)* | 0.310 | *10 % (92 %)* | 0.211 | *4 % (97 %)* |
| Chl *b*_mass_ | 0.731 | *53 % (53 %)* | 0.266 | *7 % (60 %)* | 0.525 | *28 % (88 %)* | -0.136 | *2 % (90 %)* |
| Chl *b*_area_ | 0.726 | *53 % (53 %)* | 0.410 | *17 % (70 %)* | 0.463 | *21 % (91 %)* | -0.103 | *1 % (92 %)* |
| Basal stem diameter | -0.613 | *38 % (38 %)* | -0.348 | *12 % (50 %)* | 0.482 | *23 % (73 %)* | -0.031 | *0 % (73 %)* |
| Shoot dry mass content | 0.607 | *37 % (37 %)* | 0.426 | *18 % (55 %)* | -0.506 | *26 % (81 %)* | -0.235 | *6 % (86 %)* |
| Leaf thickness | -0.562 | *32 % (32 %)* | -0.162 | *3 % (34 %)* | 0.317 | *10 % (44 %)* | 0.162 | *3 % (47 %)* |
| PNUE | 0.530 | *28 % (28 %)* | -0.769 | *59 % (87 %)* | -0.187 | *4 % (91 %)* | 0.089 | *1 % (91 %)* |
| CC_mass_ | 0.519 | *27 % (27 %)* | 0.679 | *46 % (73 %)* | 0.002 | *0 % (73 %)* | -0.081 | *1 % (74 %)* |
| P_stem_ | -0.508 | *26 % (26 %)* | 0.019 | *0 % (26 %)* | -0.102 | *1 % (27 %)* | 0.767 | *59 % (86 %)* |
| LDMC | 0.240 | *6 % (6 %)* | 0.363 | *13 % (19 %)* | -0.741 | *55 % (74 %)* | 0.102 | *1 % (75 %)* |
| P_leaf sheath_ | 0.427 | *18 % (18 %)* | -0.435 | *19 % (37 %)* | 0.198 | *4 % (41 %)* | -0.652 | *43 % (84 %)* |
| Variation Explained | 10.733 | *54 % (54 %)* | 2.857 | *14 % (68 %)* | 2.120 | *11 % (79 %)* | 1.459 | *7 % (86 %)* |

**Table S6** Discriminant analysis for genotypes based on the groups of origins (EU: native European; AM: introduced North American; AMn: native North American.). Words in red indicate miss-predicted samples.

| **Genotypes** | On the basis of origins | |
| --- | --- | --- |
|  | predicted | Prob(predicted) (%) |
| AM 115 | AM | 93.03 |
| AM 152 | EU | 93.47 |
| AM 114 | AM | 99.98 |
| AM 206 | AM | 99.88 |
| AM 199 | AM | 99.48 |
| AM 99 | AM | 99.96 |
| AM 180 | AM | 99.71 |
| AM 191 | AM | 75.62 |
| AM 186 | AM | 99.67 |
| EU 67 | EU | 98.25 |
| EU 639 | EU | 62.66 |
| EU 85 | EU | 99.97 |
| EU 801 | EU | 99.92 |
| EU 163 | EU | 98.75 |
| EU 172 | EU | 92.36 |
| AMn 204 | AMn | 100 |
| AMn 55 | AMn | 99.96 |
| AMn 130 | AMn | 100 |
| AMn 65 | AMn | 100 |
| AMn 211 | AMn | 99.06 |
| -2Log likelihood | 7.36 | |
